# Therapiemanagement des mCRPC bei Kombinationen mit PARP-Inhibitoren in der klinischen Praxis

**DOI:** 10.1055/a-2669-8781

**Published:** 2025-10-27

**Authors:** Margitta Retz, Angelika Borkowetz, Katja Wittenzellner, Heja Aga-Barfknecht, Gunhild von Amsberg

**Affiliations:** 1 Urologische Klinik und Poliklinik, TUM Universitätsklinikum Rechts der Isar, München, Deutschland; 2 39071 Klinik und Poliklinik für Urologie, Universitätsmedizin Rostock, Rostock, Deutschland; 3 42230 Medical Affairs Oncology, Pfizer Pharma GmbH, Berlin, Deutschland; 4 37734 Onkologisches Zentrum/ Martini-Klinik, Universitätsklinikum Hamburg-Eppendorf, Hamburg, Deutschland

**Keywords:** metastasiertes kastrationsresistentes Prostatakarzinom, Therapiemanagement, Nebenwirkungsmanagement, PARP-Inhibitoren, Systemtherapie, metastatic castration-resistant prostate cancer, therapy management, side-effect management, systemic therapy, PARP inhibitors

## Abstract

Innovative Therapieansätze wie die Poly-(ADP-Ribose)-Polymerase-Inhibitoren (PARPi) haben beim metastasierten kastrationsresistenten Prostatakarzinom (mCRPC), insbesondere in Kombination mit Androgenrezeptorblockern (ARPi), vielversprechende Ergebnisse gezeigt. Patienten profitieren – unabhängig von HRR-Genmutationen – von verbesserten radiologischen progressionsfreien Überlebensraten und einem längeren Gesamtüberleben. Der Erfolg der Kombinationstherapie mit PARPi und ARPi hängt maßgeblich von einem effektiven Therapie- und dem Nebenwirkungsmanagement ab. Häufige hämatologische Nebenwirkungen sind Anämie, Leukopenie und Thrombozytopenie, während bei den nicht-hämatologischen Reaktionen insbesondere Fatigue, Diarrhö, Übelkeit und Obstipation von Bedeutung sind. Neben der Basisdiagnostik und präventiven Maßnahmen können, abhängig vom Schweregrad der Nebenwirkungen, Dosisanpassungen oder Therapiepausen erforderlich sein. Bei Anämie, der häufigsten Nebenwirkung, können unterstützende Maßnahmen wie Bluttransfusionen notwendig werden, um eine optimale Patientenversorgung sicherzustellen. Dieser Leitfaden bietet Uro-Onkologen praxisorientierte Handlungsempfehlungen für den klinischen Alltag.

## Abstract (en)

Innovative therapeutic approaches, including poly(ADP-ribose)polymerase inhibitors (PARPi), have shown promising results in metastatic castration-resistant prostate cancer (mCRPC), particularly when combined with androgen receptor inhibitors (ARPi). Irrespective of HRR gene mutations, patients benefit from improved radiological progression-free survival and overall survival. The success of PARPi/ARPi combination therapy relies heavily on the effective management of both treatment administration and the associated side-effects. Common haematological side-effects include anaemia, leukopenia, and thrombocytopenia, whereas non-haematological reactions – particularly fatigue, diarrhea, nausea, and constipation – are also clinically relevant. In addition to basic diagnostics and preventive measures, dose adjustments or temporary discontinuation may be required depending on the severity of the side-effects. For anaemia, the most common side-effect, supportive measures such as blood transfusions may be necessary to ensure optimal patient care. This guide provides uro-oncologists with practical recommendations for daily clinical practice.

## Einleitung

Innovative therapeutische Ansätze wie die Poly-(ADP-Ribose)-Polymerase-Inhibitoren (PARPi) eröffnen große Chancen, sowohl das Überleben als auch die Lebensqualität von Patienten 1 * mit metastasiertem kastrationsresistenten Prostatakarzinom (mCRPC) zu verbessern. Der Erfolg dieser Behandlungsstrategien ist jedoch maßgeblich von einem effizienten Management der Therapie sowie der damit verbundenen Nebenwirkungen abhängig. Dieser Artikel bietet eine umfassende Übersicht über die potenziellen Nebenwirkungen von Kombinationstherapien mit PARPi und Androgenrezeptorblockern (ARPi) in der klinischen Praxis und stellt kompakte Handlungsanweisungen für den Uro-Onkologen zur Verfügung.

### PARPi als neue Substanzklasse beim metastasierten Prostatakarzinom

Die Aufrechterhaltung der genomischen Stabilität ist essenziell für das Überleben der Zelle, da die Akkumulation von Fehlern und Schäden unweigerlich zum Zelltod führt. Poly-(ADP-Ribose)-Polymerasen (PARPs) bilden eine Familie von Enzymen, die für die Reparatur von DNA-Schäden verantwortlich sind. Unter diesen ist PARP1 das am intensivsten untersuchte Enzym, das sowohl an der Reparatur von Einzel- und Doppelstrangbrüchen als auch an der Stabilisierung der Replikationsgabel beteiligt ist 1 .

PARPi stellen eine neue Substanzklasse in der Therapie des mCRPC dar. Sie bewirken eine Persistenz und Akkumulation von Einzelstrangbrüchen. Dies geschieht einerseits durch die direkte Hemmung der enzymatischen Aktivität von PARP, andererseits durch das sogenannte „PARP-Trapping“, bei dem PARP-Moleküle an DNA-Bindungsstellen fixiert werden. Beide Mechanismen destabilisieren die Replikationsgabeln und führen infolgedessen zur Bildung von Doppelstrangbrüchen 2 . Liegt ein Defekt in der homologen Reparatur vor, können diese Doppelstrangbrüche nicht repariert werden und sind für die Zelle letal.

Das Verständnis des molekularen Zusammenhangs zwischen dem Androgenrezeptor-Signalweg (AR-Signalweg) und den DNA-Reparaturmechanismen hat zur Anwendung des Konzepts der synthetischen Letalität zu therapeutischen Zwecken geführt 3 . Tumorzellen, bei denen wichtige Reparaturproteine für die homologe Rekombination (HRR) defekt sind, fehlen oder nicht richtig funktionieren, sind empfindlicher gegenüber Medikamenten, die Doppelstrangbrüche in der DNA begünstigen 4 . Die Hemmung des AR-Signalwegs ist dabei mit einer verminderten Expression HRR-assoziierter Gene verbunden 5 6 7 . Folge ist eine funktionelle Einschränkung der homologen Reparatur. Dies wird auch als *BRCAness-Effekt* bezeichnet 8 9 . PARPi können ihrerseits die Tumorempfindlichkeit gegenüber ARPi steigern, indem sie die AR-Transkriptionsaktivität unterbinden 10 11 . Diese beiden Mechanismen verstärken sich gegenseitig.

### PARPi-Monotherapie im mCRPC

Als Monotherapie wurden PARPi-Substanzen zunächst bei mCRPC-Patienten mit einem Defekt in den HRR-Genen untersucht. Dabei zeigte sich eine besonders hohe Aktivität bei Patienten mit einer pathogenen BRCA1/2-Alteration, während die selteneren Alterationen mit wechselndem Therapieansprechen einhergingen. So führte Olaparib als PARPi in der randomisierten Phase-III-Studie PROfound im Vergleich zu einem ARPi-Wechsel zu einem signifikanten Überlebensvorteil 12 . Basierend auf den Ergebnissen der PROfound-Studie ist Olaparib als Monotherapie bei mCRPC-Patienten mit Nachweis einer BRCA1/2-Mutation zugelassen, wenn bereits eine Vorbehandlung mit einem ARPi erfolgte.

### PARPi-/ARPi-Kombinationtherapie im mCRPC

Drei Phase-III-Studien untersuchten die Wirksamkeit und Sicherheit einer PARPi-/ARPi-Kombination beim mCRPC. In der TALAPRO-2-Studie erfolgte eine Kombination des PARPi Talazoparib mit Enzalutamid, während in der MAGNITUDE-Studie Niraparib zusammen mit Abirateron/Prednison und in der PROpel-Studie Olaparib ebenfalls in Kombination mit Abirateron/Prednison oder Prednisolon (PP) untersucht wurde ( Tab. 1 ) 13 14 15 . In allen 3 Studien wurden Patienten in der Erstlinie des mCRPC eingeschlossen. Die Einschlusskriterien erlaubten im Stadium des metastasierten hormonsensitiven Prostatakarzinoms (mHSPC) die Anwendung einer Docetaxel-Chemotherapie. Je nach Studie war zudem eine vorherige ARPi-Therapie zulässig, die jedoch nur wenige Studienpatienten erhielten. Prospektive Studiendaten zu einer Vortherapie mit einer kombinierten Hormontherapie oder Tripletherapie im mHSPC fehlen derzeit. Primärer Endpunkt war bei allen Studien das radiologisch progressionsfreie Überleben (rPFS). Die Studien unterscheiden sich hauptsächlich im Studiendesign in Bezug auf den Zeitpunkt der Mutationsanalyse sowie in den untersuchten Kohorten. In der PROpel-Studie wurden alle mCRPC-Patienten unabhängig von möglichen HRR-Mutationen in eine sogenannte Allcomer-Kohorte aufgenommen. Die Sequenzierung der HRR-Gene erfolgte im Rahmen einer retrospektiven Auswertung. In der MAGNITUDE-Studie hingegen wurde prospektiv, also vor Randomisierung, auf vorhandene HRR-Mutationen getestet. Hier erfolgte demnach bereits zu Beginn der Therapie eine klare Differenzierung zwischen einer HRR-defizienten Kohorte und einer Gruppe ohne HRR-Mutation. Die TALAPRO-2-Studie untersuchte 2 Kohorten, eine Allcomer- und eine HRR-defiziente Gruppe, die prospektiv molekulargenetisch untersucht wurden. In die Allcomer-Kohorte wurden ebenfalls Patienten unabhängig von ihrem HRR-Status eingeschlossen, während in der HRR-defizienten Kohorte ausschließlich Patienten mit HRR-Mutation berücksichtigt wurden.

**Table:** **Tab. 1** Übersicht der Zulassungsstudien TALAPRO-2, MAGNITUDE und PROpel bezüglich Studiendesign, Endpunkte und Zulassung 13 14 15 16 17 18 19 20 *.

Studie	TALAPRO-2	MAGNITUDE	PROpel
**experimentelle Therapie**	Enzalutamid + Talazoparib	Abirateron/PP + Niraparib	Abirateron/PP + Olaparib
**Vergleichstherapie**	Enzalutamid	Abirateron/PP	Abirateron/PP
**Studienpopulation**	„Allcomer“ und HRR-angereicherte Kohorte	HRR-negative (vorzeitig gestoppt) und HRR-positive Kohorte	„Allcomer“
**primärer Endpunkt**	rPFS (BICR)	rPFS (BICR)	rPFS (Investigator)
**erlaubte Vortherapie (% vorbehandelter Patienten)**	Docetaxel (HSPC) (Kohorte 1: 21%; Kohorte 2: 28%) Abirateron (nmCRPC/HSPC) (Kohorte 1: 5%; Kohorte 2: 8%)	Docetaxel (HSPC) (19,3%) ARPi außer Abirateron (nmCRPC/HSPC) (3,8%) Abirateron (mCRPC ≤4 Monate) (23,6%)	Docetaxel (HSPC) (22,6%) kein ARPi 12 Monate vor Studienstart
**rPFS („Allcomer“) HR (95%-KI);** p-Wert	0,63 (0,51–0,78); p<0,0001**	NA	0,66 (0,54–0,81); p<0,001**
**rPFS (HRR+) HR (95%-KI);** p-Wert	0,45 (0,33; 0,61); p<0,0001**	0,73 (0,56–0,96); p=0,022	0,50 (0,34–0,73)
**rPFS (BRCA1/2+) HR (95%-KI);** p-Wert	0,20 (0,11–0,36); p<0,0001	0,53 (0,36-0,79); p=0,001	0,23 (0,12–0,43)
**rPFS (HRR–) HR (95%-KI);** p-Wert	0,66 (0,49–0,91); p=0,0092	NA	0,76 (0,60–0,97)
**OS („Allcomer“) HR (95%-KI);** p-Wert	0,80 (0,66-0,96); p=0,016**	NA	0,81 (0,67–1,00); p=0,054**
**OS (HRR+) HR (95%-KI);** p-Wert	0,62 (0,48–0,81); p=0,0005**	0,93 (0,72-1,20)	0,66 (0,45–0,95)
**OS (BRCA1/2+) HR (95%-KI);** p-Wert	0,50 (0,32-0,78); p=0,0017	0,79 (0,55–1,12); p=0,18	0,29 (0,14–0,56)
**OS (HRR–) HR (95%-KI);** p-Wert	0,78 (0,58–1,05); p=0,10	NA	0,89 (0,7–1,14)
**Zulassung**	Talazoparib wird in Kombination mit Enzalutamid zur Behandlung von mCRPC-Patienten angewendet, bei denen eine Chemotherapie klinisch nicht indiziert ist.	Das Kombinationspräparat aus Niraparib und Abirateron wird angewendet mit PP zur Behandlung von mCRPC-Patienten mit BRCA1/2-Mutationen (in der Keimbahn und/oder somatisch), bei denen eine Chemotherapie nicht klinisch indiziert ist.	Olaparib wird in Kombination mit Abirateron und PP zur Behandlung von mCRPC-Patienten angewendet, bei denen eine Chemotherapie klinisch nicht indiziert ist.
ARPi=Androgenrezeptorblocker, BICR=Blinded Independent Central Review, BRCA=Breast Cancer Gene, HR=Hazard Ratio, HRR=homologe Rekombinationsreparatur, HSPC=hormonsensitives Prostatakarzinom, KI=Konfidenzintervall, NA=nicht angegeben, nmCRPC=nicht metastasiertes kastrationsresistentes Prostatakarzinom, mCRPC=metastasiertes kastrationsresistentes Prostatakarzinom, OS=Gesamtüberleben, PP=Prednison oder Prednisolon, rPFS=radiologisches progressionsfreies Überleben; *kein direkter Vergleich zwischen den Zulassungsstudien erlaubt. Darstellung rein zu informativen Zwecken; ** alpha-geschützter p-Wert			

In allen Studien war die Kombinationstherapie aus ARPi und PARPi mit einer signifikanten Verbesserung des rPFS bei Patienten mit einer BRCA1/2-Mutation im Vergleich zur ARPi-Monotherapie verbunden. Zudem zeigten auch die Gruppen mit einer HRR-Mutation, die nicht die BRCA1/2-Gene betraf, deutliche Vorteile zugunsten der PARPi-/ARPi-Kombinationen. In der TALAPRO-2- und PROpel-Studie profitierten auch Patienten ohne HRR-Mutation in der Allcomer-Gruppe mit der PARPi-/ARPi-Kombination im rPFS mit einer Hazard Ratio von 0,66 bzw. 0,76 ( Tab. 1 ). Das Gesamtüberleben (OS) wurde in der TALAPRO-2-Studie als einzigem PARPi-/ARPi-Kombinationsansatz mit Talazoparib und Enzalutamid signifikant verbessert, sowohl in der HRR-defizienten Kohorte als auch in der Allcomer-Gruppe. Die Interimsanalyse der MAGNITUDE-Studie ergab in der HRR-negativen Kohorte keinen Vorteil von Niraparib plus Abirateron/PP gegenüber Abirateron/PP allein, weshalb diese Kohorte frühzeitig gestoppt wurde. Eine Übersicht der PARPi-/ARPi-Zulassungsstudien TALAPRO-2, MAGNITUDE und PROpel bezüglich Studiendesign, Endpunkte und Zulassung findet sich in Tab. 1 .

Die Bewertung der Effektivitätsdaten zwischen der amerikanischen und europäischen Zulassungsbehörde führte zu unterschiedlichen Zulassungen, mit einer Beschränkung auf HRR-/BRCA-Mutationen in den USA. Auch in Europa führten die Effektivitätsdaten der 3 pivotalen Studien zu unterschiedlichen Arzneimittelzulassungen: Die Kombinationstherapie aus Niraparib und Abirateron/PP ist ausschließlich für mCRPC-Patienten mit einer BRCA1/2-Mutation zugelassen 21 . Dagegen sind die Kombinationen aus Talazoparib und Enzalutamid sowie Olaparib und Abirateron/PP unabhängig vom Mutationsstatus im mCRPC-Stadium zugelassen ( Tab. 1 ) 22 23 .

## Dosierungen, Dosisreduktionen und Wechselwirkungen

### Dosierungen und Dosisanpassungen

Die Standarddosierungen der PARPi-/ARPi-Kombinationen sind in Tab. 2 aufgelistet. Nebenwirkungen durch PARPi-/ARPi-Kombinationen werden überwiegend durch Dosisanpassungen und, wenn notwendig, durch Therapieunterbrechung kontrolliert. Bei PARPi-induzierten schweren Nebenwirkungen sollte im Allgemeinen eine Therapieunterbrechung erfolgen, bis die Symptome abgeklungen sind. Anschließend kann die Therapie mit der nächstniedrigeren Dosis fortgeführt werden 21 22 23 . Für den PARPi Talazoparib stehen beim mCRPC Hartkapseln mit 0,25 und 0,1 mg zur Verfügung 22 . Olaparib-Filmtabletten enthalten entweder 150 oder 100 mg Wirkstoff 23 . Bei Niraparib ist zu beachten, dass die Filmtabletten als fixe Kombination den PARPi und Abirateron zugleich enthalten. Es stehen Wirkstärken der Kombination Niraparib/Abirateron mit 100 mg/500 mg und 50 mg/500 mg zur Verfügung 21 .

Die Therapie mit Enzalutamid sollte bei nicht tolerierbaren oder schweren Toxizitäten für eine Woche unterbrochen werden. Nach Besserung der Symptome kann die Behandlung mit unveränderter (160 mg/Tag) oder reduzierter Dosis (120 bzw. 80 mg/Tag) fortgesetzt werden 24 . Schwere Abirateron-bedingte Nebenwirkungen erfordern eine Unterbrechung der Behandlung, bis die Symptome abgeklungen oder auf den Ausgangswert zurückgegangen sind. Die Behandlung mit Abirateron kann anschließend in gleicher Dosis wieder aufgenommen werden 25 . In Tab. 2 sind die empfohlenen Anfangsdosierungen, mögliche Dosisreduktionen und Halbwertszeiten der zugelassenen PARPi-/ARPi-Kombinationstherapien zusammengefasst ( Tab. 2 ) 21 22 23 24 25 .

**Table:** **Tab. 2** Übersicht der Dosierungsmöglichkeiten, Halbwertszeiten und empfohlene Abstände der Blutbildkontrollen der verschiedenen Kombinationen mit PARPi und ARPi 21 22 23 24 25 .

	Kombination aus Talazoparib und Enzalutamid		Kombinationspräparat aus Niraparib/Abirateron mit Prednison/Prednisolon		Kombination aus Olaparib, Abirateron und Prednison/Prednisolon		
	Talazoparib HWZ: 90 Stunden	Enzalutamid HWZ: 5,8 Tage	Kombipräparat N + A HWZ: N=62 Stunden A=20 Stunden	PP	Olaparib HWZ: 15 Stunden	Abirateronacetat HWZ: 15 Stunden	PP
**Anfangsdosierung pro Tag**	0,50 mg ≙ 1-mal pro Tag 2 Tabl. mit 0,25 mg	160 mg ≙ 1-mal pro Tag 4 Tabl. mit 40 mg	200 mg N/1000 mg A ≙ 1-mal pro Tag 2 Tabl. mit 100 mg N/500 mg A	10 mg ≙ 2-mal pro Tag 1 Tabl. mit 5 mg	600 mg ≙ 2-mal pro Tag 2 Tabl. mit 150 mg	1000 mg ≙ 1-mal pro Tag 2 Tabl. mit 500 mg	10 mg ≙ 2-mal pro Tag 1 Tabl. mit 5 mg
**1. Dosisreduktion**	0,35 mg ≙ 1-mal pro Tag 1 Tabl. mit 0,25 mg und 1 Tabl. mit 0,1 mg	120 mg ≙ 1-mal pro Tag 3 Tabl. mit 40 mg	100 mg N/1000 mg A ≙ 1-mal pro Tag 2 Tabl. mit 50 mg N/500 mg A		500 mg ≙ 2-mal pro Tag 1 Tabl. mit 150 mg und 1 Tabl. mit 100 mg		
**2. Dosisreduktion**	0,25 mg ≙ 1-mal pro Tag 1 Tabl. mit 0,25 mg	80 mg ≙ 1-mal pro Tag 2 Tabl. mit 40 mg			400 mg ≙ 2-mal pro Tag 2 Tabl. mit 100 mg		
**3. Dosisreduktion**	0,1 mg ≙ 1-mal pro Tag 1 Tabl. mit 0,1 mg						
**Blutbildkontrollen**	vor Therapiebeginn ab 1. Monat: monatlich		vor Therapiebeginn 1. Monat: wöchentlich 2. und 3. Monat: 14-tägig 4.–12. Monat: monatlich ab 12. Monat: alle 2 Monate		vor Therapiebeginn 1.–12. Monat: monatlich ab 12. Monat: periodische Abstände		
A=Abirateron, N=Niraparib, PP=Prednison oder Prednisolon, Tabl.=Tablette, HWZ=Halbwertszeit							

#### Dosisanpassung bei Nierenfunktionsstörungen

Bei leichter Nierenfunktionsstörung sind keine Dosisanpassungen von PARPi erforderlich 21 22 23 . Besteht eine mittelschwere Niereninsuffizienz mit einer glomerulären Filtrationsrate [GFR] von 30–59 ml/min, sollte eine Dosisreduktion bei Talazoparib auf 0,35 und Olaparib auf 400 mg täglich erfolgen 22 23 . Bei Niraparib ist keine Dosisanpassung notwendig, allerdings sollten die Patienten engmaschig überwacht werden 21 . Bei schwerer Niereninsuffizienz mit einer GFR von 15–29 ml/min sollte die Talazoparib-Dosis auf 0,25 mg täglich reduziert werden 22 . Die Anwendung von Olaparib wird in diesem Fall nicht empfohlen, während bei Niraparib der Nutzen die potenziellen Risiken überwiegen sollte 21 23 .

#### Dosisanpassung bei Leberfunktionsstörungen

Leichte und mittelschwere Leberinsuffizienz erfordert keine Anpassungen der PARPi-Dosis. Die Kombinationstherapien mit Talazoparib, Olaparib und Niraparib wurden nicht an Patienten mit schwerer Leberfunktionseinschränkung der Child-Pugh-Klassifikation C getestet und werden daher entsprechend der Fachinformation nicht empfohlen 21 22 23 .

### Wechselwirkungen

Arzneimittelinteraktionen können die Konzentration gleichzeitig eingesetzter Medikamente verändern, wodurch ihre beabsichtigte Wirkung verstärkt, reduziert oder aufgehoben werden kann. Sowohl PARPi- als auch ARPi-basierte Kombinationen zeigen potenzielle Medikamenteninteraktionen. Einerseits können ARPis Enzyme wie CYP3A4 oder CYP2C9 entweder verstärkend oder hemmend beeinflussen, andererseits können diese Enzyme auch die Aktivität der ARPis in unterschiedlichem Maße modulieren 26 . Ein wichtiger klinischer Aspekt ist, dass bei Übelkeit während einer PARPi-/ARPi-Kombinationstherapie die Antiemetika Aprepitant und Fosaprepitant aufgrund möglicher Wechselwirkungen vermieden werden sollten. Zudem weisen die häufig bei Thrombosen eingesetzten neuen oralen Antikoagulanzien sowie Clopidogrel, das zur Thrombozytenhemmung bei arteriellen Verschlüssen und Durchblutungsstörungen eingesetzt wird, zahlreiche Interaktionen mit ARPis und PARPis auf. In diesem Zusammenhang ist besondere Vorsicht geboten, weshalb Apotheker sowie Fachkollegen aus der Hämostaseologie hinzugezogen werden sollten. Bei Patienten mit Polypharmazie und komplexer Komedikation erfordert nicht jede moderate oder schwere Wechselwirkung zwingend die Beendigung der Medikation. Es ist entscheidend, die Konsequenzen der Interaktion sorgfältig zu prüfen. Hierzu sollte ein pharmazeutisches Konsil erstellt oder zumindest die verfügbaren Interaktionsrechner im Internet (z.B. www.drugs.com ) genutzt werden.

Ein beispielhafter Therapiebogen der Urologischen Klinik und Poliklinik, TUM Universitätsklinikum Rechts der Isar der Technischen Universität München, ist über einen QR-Code zugänglich und kann in der klinischen Praxis von Nutzen sein (Kasten QR).

## Nebenwirkungen

Die potenziellen Nebenwirkungen und das Management der PARPi-/ARPi-Kombinationen bei mCRPC-Patienten stellen eine Herausforderung in der klinischen Praxis dar. Die folgenden Übersichten zu den häufigsten Nebenwirkungen sollen das klinische Management erleichtern.

### Nebenwirkungen unter ARPi-Monotherapie

Zu den häufigsten Nebenwirkungen von Enzalutamid zählen unter anderem Fatigue, Asthenie, Hitzewallungen, Hypertonie, Frakturen und Stürze. Außerdem sollte auf weitere wichtige Nebenwirkungen wie ischämische Herzerkrankungen oder Krampfanfälle geachtet werden 24 . Unter Abirateron treten bei einem höheren Anteil der Patienten periphere Ödeme, Hypokaliämie, Hypertonie, Harnwegsinfektionen sowie Anstiege der Alaninaminotransferase (ALAT) und/oder Aspartataminotransferase (ASAT) auf. Weitere relevante Nebenwirkungen wie Herzerkrankungen, Hepatotoxizität, Frakturen und allergische Alveolitis sind zu beachten 25 .

### Nebenwirkungen unter PARPi-Monotherapie

Zu den häufigsten Nebenwirkungen der PARPi gehört eine Beeinträchtigung der Hämatopoese. Die Hemmung der PARP-Aktivität führt als unerwünschte Wirkung zu einer Schädigung schnell proliferierender Vorläuferzellen in der Hämatopoese, die stark auf intakte DNA-Reparaturmechanismen angewiesen sind. Zusätzlich können irreparable DNA-Schäden einen Zellzyklusstopp oder eine Apoptose induzieren, was die Regeneration der Hämatopoese weiter beeinträchtigt 3 4 . Klinisch äußern sich diese Störungen der Hämatopoese in Form von Anämie sowie Leukopenie bzw. Neutropenie und/oder Thrombozytopenie 4 10 . Diese Nebenwirkungen treten typischerweise in den ersten Monaten der Behandlung auf und nehmen im weiteren Verlauf häufig ab 8 11 . Talazoparib zeigt die stärkste PARP-Trapping-Aktivität, was die vergleichsweise höhere Hämatotoxizität unter diesem Wirkstoff erklären könnte 4 .

### Nebenwirkung unter PARPi-/ARPi-Kombinationstherapie

Unter der Kombinationstherapie aus PARPi und ARPi dominieren vor allem die PARPi-typischen hämatologischen Nebenwirkungen ( Tab. 3 ) 13 14 15 . Zu den nicht hämatologischen Nebenwirkungen der PARPi-/ARPi-Kombination zählen Fatigue, Schwindel, Arthralgien sowie kardiovaskuläre Beschwerden einschließlich Hypertonie. Darüber hinaus sind auch gastrointestinale Nebenwirkungen wie Übelkeit, Erbrechen, Diarrhö und Obstipation bekannt. Die substanzspezifischen Unterschiede in Häufigkeit und Schweregrad sind in den Zulassungsstudien dokumentiert und werden nach betroffenen Organsystemen kategorisiert ( Tab. 3 ) 13 14 15 .

**Table:** **Tab. 3** Auswahl der häufigsten Nebenwirkungen unter der Kombination mit PARPi und ARPi 13 14 15 .

Systemorganklasse	Nebenwirkungen	TALAPRO-2 Talazoparib/Enzalutamid		MAGNITUDE Niraparib/Abirateron/PP		PROpel Olaparib/Abirateron/PP	
		alle Grade (%)	Grad ≥3 (%)	alle Grade (%)	Grad ≥3 (%)	alle Grade (%)	Grad ≥3 (%)
Erkrankungen des Blutes und Lymphsystems	Anämie	66	46	46	30	46	15
	Leukopenie	22	6	10	2	NA	NA
	Neutropenie	36	18	14	7	NA	NA
	Thrombozytopenie	25	7	21	7	NA	NA
Erkrankungen des Gastrointestinaltraktes und Ernährungsstörungen	Diarrhö	14	<1	NA	NA	17	<1
	Erbrechen	NA	NA	13	<1	13	1
	Obstipation	18	<1	31	0	17	0
	Übelkeit	21	<1	24	<1	28	<1
	verminderter Appetit	22	1	14	<1	15	1
allgemeine Erkrankungen	Asthenie	14	<3	16	<1	37	2
	Fatigue	34	4	26	3		
	periphere Ödeme	11	0	NA	NA	10	0
Skelettmuskulatur-, Bindegewebs- und Knochenerkrankungen	Arthralgie	15	<1	13	<1	13	0
	Rückenschmerzen	22	3	15	2	17	<1
Gefäßerkrankungen	Hypertonie	14	5	31	15	13	4
Erkrankungen des Nervensystems	Schwindel	12	1	11	<1	11	0
Erkrankungen der Atemwege, des Brustraums und Mediastinums	Dyspnoe	10	<1	16	2	NA	NA
eingeschlossen wurden Nebenwirkungen Grad 1–4, die bei mindestens 2 Kombinationsregimen und bei mindestens 10% der Studienpopulation aufgetreten sind. PP= Prednison oder Prednisolon, NA=nicht angegeben oder Auftreten <10%							

Die unter der Kombination von PARPi und ARPi auftretenden Nebenwirkungen von besonderem Interesse sind das myelodysplastische Syndrom (MDS) und die akute myeloische Leukämie (AML). In den Zulassungsstudien wurden diese mit einer Inzidenz von 0 bzw. <1% sehr selten beschrieben. Ein möglicher Zusammenhang zwischen der Anwendung von PARPi und dem Auftreten von MDS bzw. AML wurde insbesondere bei Frauen mit fortgeschrittenem Ovarialkarzinom und einer BRCA1/2-Keimbahnmutation diskutiert, ohne dass eine direkte Assoziation nachgewiesen werden konnte. Eine Auswertung nach Markteinführung zeigte eine AML/MDS-Rate von 0,7% (n=88/12823) unter/nach PARPi-Behandlung mit einer mutmaßlich höheren Inzidenz nach Vorbehandlung mit einer Chemotherapie 27 . Bei älteren mCRPC-Patienten und solchen mit BRCA1/2-Mutationen ist das Risiko für die Entwicklung eines MDS oder einer AML unter PARPi-Therapie aufgrund der zu erwartenden kürzeren Lebenszeit somit als vernachlässigbar anzusehen. In der Patientenaufklärung sollte dieses Risiko im Rahmen der Nutzen-Risiko-Abwägung eine untergeordnete Rolle spielen. Für die klinische Praxis sind jedoch thromboembolische Ereignisse von größerer Relevanz. Diese wurden in den Studien TALAPRO-2 und MAGNITUDE mit einer Inzidenz von 2–4% und in der PROpel-Studie mit 11% signifikant häufiger beobachtet 13 14 15 .

## Management der häufigsten Nebenwirkungen bei PARPi-/ARPi-Therapien

Für einen möglichst ausgeprägten Behandlungserfolg mit PARPi-/ARPi-Kombinationen ist ein effektives Therapiemanagement entscheidend. Häufige hämatologische Nebenwirkungen umfassen Anämie, Leukopenie und Thrombozytopenie. Zu den wichtigen nicht hämatologischen Nebenwirkungen gehören Fatigue, Diarrhö, Übelkeit und Obstipation 13 14 15 .

### Anämie 28

#### Definition

**Abb. 1:**
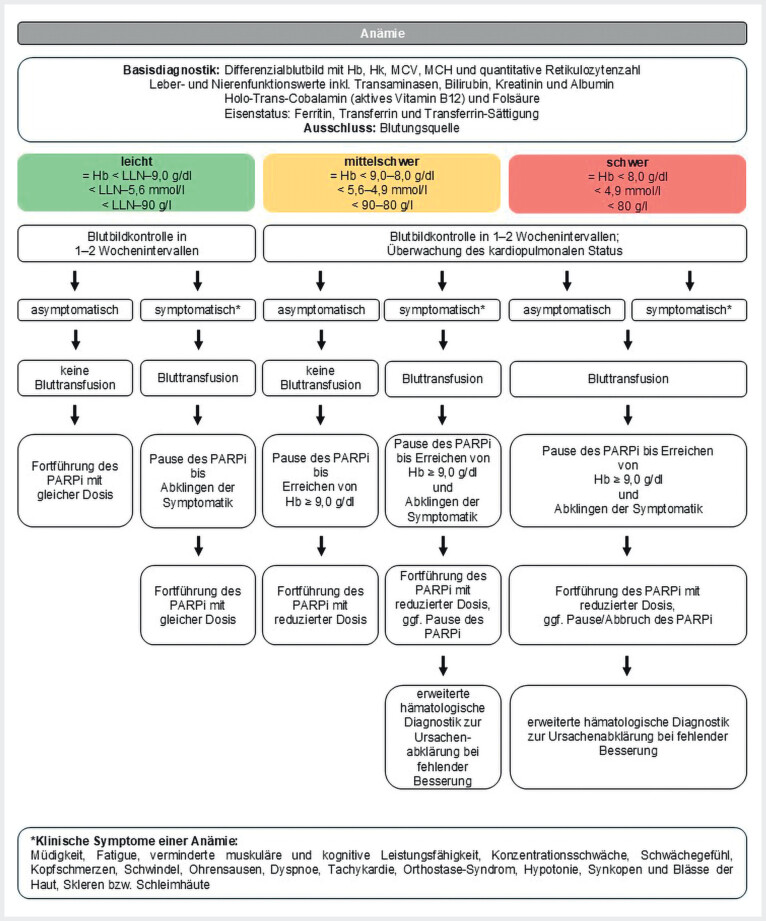
Schema zum Therapiemanagement bei Auftreten einer Anämie 28 . Hb=Hämoglobin, Hk=Hämatokrit, LLN=Lower Limit of Normal, MCV=mittleres korpuskuläres Volumen, MCH=mittlerer korpuskulärer Hämoglobingehalt, PARPi=PARP-Inhibitor

Unter Anämie wird eine Verminderung der Hämoglobinkonzentration und/oder des Hämatokrits im peripheren Blut verstanden. Entsprechend den Kriterien der Weltgesundheitsorganisation ist der untere Referenzwert des Hämoglobins bei mitteleuropäischen Erwachsenen, abhängig vom Alter, bei nicht schwangeren Frauen mit 12 g/dl (7,45 mmol/l) und bei Männern mit 13 g/dl (8,07 mmol/l) definiert. Eine Anämie kann durch die Tumorerkrankung selbst sowie durch eine Therapie (System- oder Radiotherapie bzw. Operation) verursacht werden. Daneben müssen auch andere klassische Ursachen wie z.B. ein Vitamin-B _12_ -Mangel oder Eisenmangelanämie bedacht werden (s. Basisdiagnostik, Abb. 1 ).

#### Symptome

Die Symptome der Anämie sind vielfältig und können alle Organsysteme betreffen. Diese können sich äußern als Müdigkeit, Schwindel, Ohrensausen, Tachykardie, Dyspnoe und Hypotonie sowie eine verminderte muskuläre und kognitive Leistungsfähigkeit. Typische Untersuchungsbefunde sind blasse Haut, Skleren und Schleimhäute ( Abb. 1 ).

#### Diagnostik vor Therapieeinleitung mit PARPi

Vor Beginn einer Therapie ist ein Hämoglobinwert von mindestens 9 g/dl (5,6 mmol/l) wünschenswert. Bei nachgewiesener Anämie ist eine Basisdiagnostik notwendig, wie in Abb. 1 dargestellt. Darüber hinaus sollte eine mögliche Blutungsquelle ausgeschlossen werden, sofern es klinische Anhaltspunkte für eine Blutung gibt. Eine symptomatische Anämie ist in jedem Fall zu behandeln.

#### Management von Nebenwirkungen unter PARPi-Therapie

Je nach Schweregrad der Anämie und dem Ausmaß der klinischen Symptomatik sollten bei einer PARPi-assoziierten Anämie Bluttransfusionen als unmittelbare therapeutische Maßnahme in Erwägung gezogen werden. Zudem wird empfohlen, die PARPi-Therapie vorübergehend zu pausieren, bis die Hämoglobinkonzentration auf mindestens 9,0 g/dl (5,6 mmol/l) angestiegen ist und die Anämiesymptome abgeklungen sind. Die Zulassung von erythropoesestimulierenden Substanzen (ESA), wie Erythropoetin, ist auf spezielle klinische Situationen beschränkt. Dazu zählen die Behandlung der symptomatischen Anämie bei chronischer Niereninsuffizienz sowie die Behandlung der Anämie bei Erwachsenen mit soliden Tumoren, malignen Lymphomen oder multiplem Myelom, die eine Chemotherapie erhalten und bei denen aufgrund des Allgemeinzustands ein Transfusionsrisiko besteht. Aus diesem Grund wurde in der Publikation auf die Empfehlung zur Anwendung von ESA außerhalb des zugelassenen Indikationsbereichs für Uro-Onkologen verzichtet. Besteht die Anämie trotz adäquatem Nebenwirkungsmanagement weiterhin, ist eine weiterführende hämatologische Abklärung indiziert ( Abb. 1 ).

### Thrombozytopenie 29 30

#### Definition

**Abb. 2:**
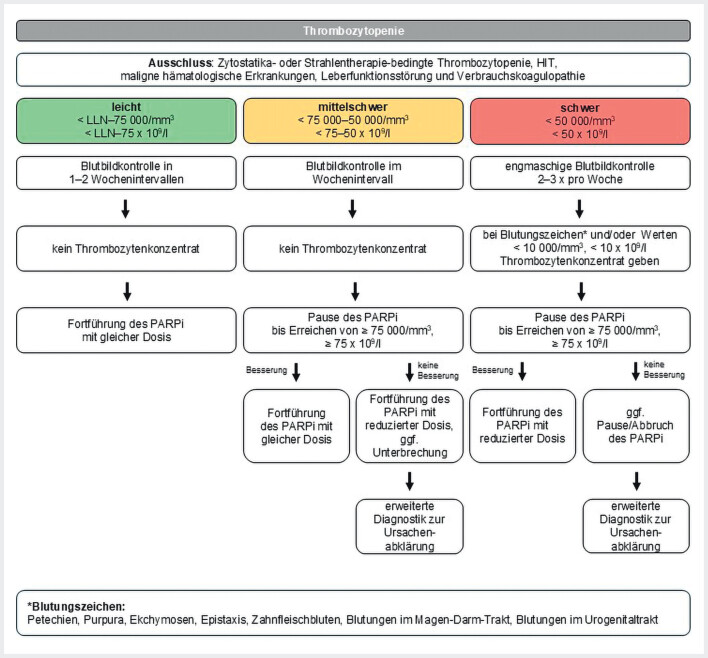
Schema zum Therapiemanagement bei Auftreten einer Thrombozytopenie 29 30 . HIT=Heparin-induzierte Thrombozytopenie, LLN=Lower Limit of Normal, PARPi=PARP-Inhibitor

Eine Thrombozytopenie liegt vor, wenn die Zahl der Thrombozyten auf <150.000/mm ^3^ (<150 × 10 ^9^ /l) sinkt. Werte zwischen 100.000 und 150.000 Thrombozyten/mm ^3^ (100–150 × 10 ^9^ /l) erfordern normalerweise keine Behandlung und sind keinem Schweregrad zugeordnet. Es werden leichte (<100.000–75.000/mm ^3^ , 100–75 × 10 ^9^ /l), mittelschwere (<75.000–50.000/mm ^3^ , <75–50 × 10 ^9^ /l), schwere (<50.000–25.000/mm ^3^ , <50–25 × 10 ^9^ /l) und sehr schwere Formen (<25 000/mm ^3^ , <25 × 10 ^9^ /l) unterschieden ( Abb. 2 ).

#### Diagnostik vor Therapieeinleitung mit PARPi

Vor Beginn einer Therapie mit einem PARPi sollten andere Ursachen für eine bestehende Thrombozytopenie ausgeschlossen werden. Hierzu gehören unter anderem Thrombozytopenien durch Zytostatika- oder Strahlentherapie, heparininduzierte Thrombozytopenie sowie maligne hämatologische Erkrankungen, Leberfunktionsstörungen oder Verbrauchskoagulopathien ( Abb. 2 ).

#### Management von Nebenwirkungen unter PARPi-Therapie

Je nach Ausprägung der Thrombozytopenie sollte der PARPi pausiert und wenn notwendig mit nächstniedrigerer Dosis fortgeführt werden. Bei schwerer Thrombozythopenie mit Werten <10.000/mm ^3^ (<10 × 10 ^9^ /l) und/oder klinisch relevanten Blutungszeichen ist die Gabe eines Thrombozytenkonzentrats indiziert. Bei sturzgefährdeten Patienten oder Vorliegen schwerer Infektionen ist eine Transfusion schon bei einem Thrombozytenwert <20.000/mm ^3^ (<20 × 10 ^9^ /l) zu erwägen. Besteht die Thrombozytopenie trotz adäquatem Nebenwirkungsmanagement weiterhin, ist eine weiterführende Abklärung indiziert ( Abb. 2 ).

### Neutropenie 31

#### Definition

**Abb. 3:**
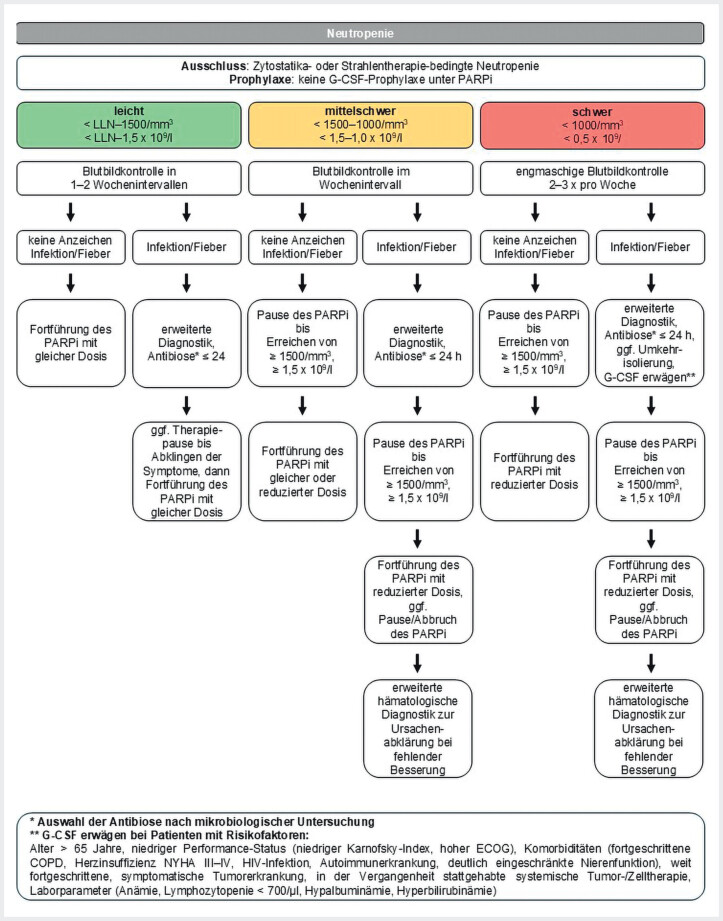
Schema zum Therapiemanagement bei Auftreten einer Neutropenie 28 31 . G-CSF=Granulozyten-Kolonie-stimulierende Faktoren, LLN=Lower Limit of Normal, PARPi=PARP-Inhibitor

Eine Neutropenie (neutrophile Granulozytopenie) liegt vor, wenn die Neutrophilenzahl <2000/mm ^3^ (<2 × 10 ^9^ /l ) beträgt. Sie wird unterteilt in leicht mit Neutrophilen <2000–1500/mm ^3^ (<2–1,5 × 10 ^9^ /l), mittelschwer <1500–1000/mm ^3^ (<1,5–1 × 10 ^9^ /l), schwer <1000–500/mm ^3^ (<1–0,5 × 10 ^9^ /l) und lebensbedrohlich <500/mm ^3^ (<0,5 × 10 ^9^ /l) ( Abb. 3 ). Eine Neutropenie sowie eine daraus resultierende febrile Neutropenie (FN) sind bedeutsame Faktoren für Morbidität und Mortalität nach zytotoxischer Therapie 28 32 . Dabei versteht man unter einer febrilen Neutropenie einen Abfall der neutrophilen Granulozyten auf <500/µl (<0,5 × 10 ^9^ /l) bzw. <1000/µl (<1 × 10 ^9^ /l) mit einem vorhersehbaren Abfall auf <500/µl (<0,5 × 10 ^9^ /l) in den nächsten beiden Tagen sowie einmalig eine Körpertemperatur >38,3 oder >38°C über mindestens 1 Stunde bzw. 2-malig im Abstand von 12 Stunden.

#### Diagnostik vor Therapieeinleitung mit PARPi

Vor Beginn der Therapie mit einem PARPi sollten eine zytostatika- und strahlentherapiebedingte Neutropenie ausgeschlossen werden. Eine prophylaktische Gabe von Granulozyten-Kolonie-stimulierenden Faktoren (G-CSF) ist bei PARPis nicht indiziert ( Abb. 3 ), da das Risiko einer febrilen Neutropenie unter 10% liegt 33 .

#### Management von Nebenwirkungen unter PARPi-Therapie

Bei Anzeichen einer febrilen Neutropenie sollte unabhängig vom Schweregrad eine erweiterte Diagnostik einschließlich einer Fokussuche mit Blut- und Urinkulturen durchgeführt und schnellstmöglich eine Antibiose gestartet werden. In Abhängigkeit der Risikofaktoren wie Tumorlast, Patientenalter, Hypotonie und Komorbiditäten entsprechend den Kriterien der Multinational Association of Supportive Care in Cancer (MASCC) muss eine Hospitalisierung erfolgen 34 . Die Auswahl der Antibiose sollte optimalerweise mithilfe einer mikrobiologischen Beratung erfolgen. Zusätzlich wird je nach Ausprägung der Neutropenie eine PARPi-Therapiepause empfohlen, bis die Werte auf ≥ 1500/mm ^3^ (≥ 1,5 × 10 ^9^ /l) angestiegen und die Symptome abgeklungen sind. Bei einer schweren febrilen Neutropenie kann gegebenenfalls zusätzlich eine Umkehrisolierung sowie bei Patienten mit Risikofaktoren eine G-CSF-Gabe erwogen werden. Besteht die Neutropenie trotz adäquatem Nebenwirkungsmanagement weiterhin, ist eine weiterführende hämatologische Abklärung indiziert ( Abb. 3 ).

### Fatigue 30 31 35 36

#### Definition

**Abb. 4:**
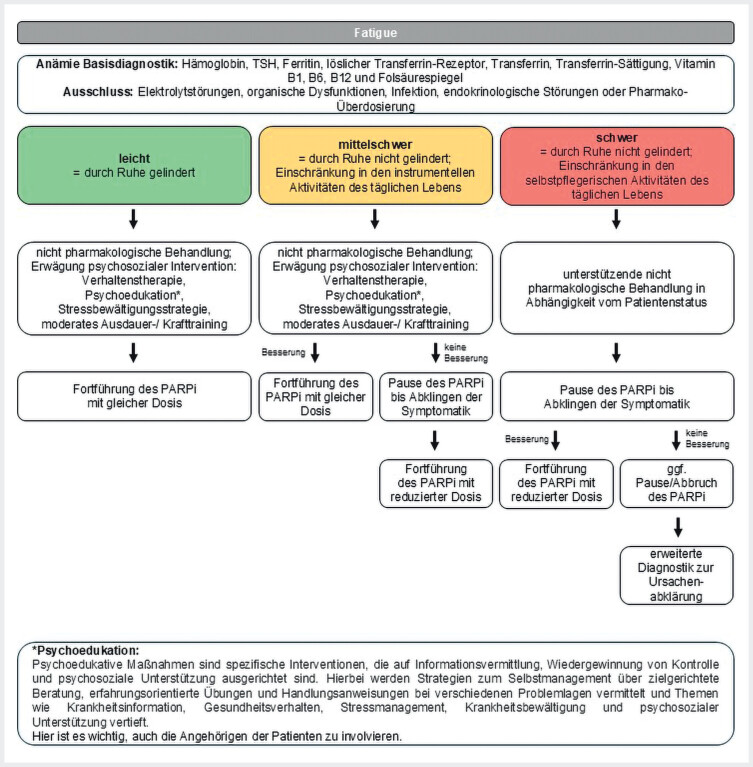
Schema zum Therapiemanagement bei Auftreten einer Fatigue 30 31 35 36 37 38 . PARPi=PARP-Inhibitor

Die außerordentliche Erschöpfung (Fatigue) äußert sich als belastendes Gefühl körperlicher, emotionaler und kognitiver Müdigkeit und ist nicht mit zuvor ausgeführten Aktivitäten verknüpft. Die tumorassoziierte Fatigue wird durch begleitende somatische oder psychische Erkrankungen sowie durch veränderte Verhaltensfaktoren wie etwa Bewegungsmangel und therapeutische Maßnahmen mitbeeinflusst. Sie führt zu einer erheblichen Beeinträchtigung sowohl gewohnter körperlicher als auch geistiger Funktionen. Differenzialdiagnostisch kommen Anämie, Schlafstörungen, Elektrolytverschiebungen, Schilddrüsenfunktionsstörungen, aber auch bestehende depressive Störungen in Betracht. Validierte Fragebögen können die Anamnese unterstützen (s. Diagnostik). Eine Depression ist von der Fatigue abzugrenzen und im Zweifel psychiatrisch fachärztlich abzuklären. Bei der milden Fatigue können sich die Betroffenen durch Ausruhen noch erholen. Das ist bei mittelschweren Symptomen nicht mehr erreichbar und instrumentelle Aktivitäten des Alltags sind nur eingeschränkt möglich. Bei schwerer Fatigue sind die Möglichkeiten zur Selbstversorgung eingeschränkt ( Abb. 4 ).

#### Diagnostik vor Therapieeinleitung mit PARPi

*Z* u Beginn der Therapie ist eine Evaluierung der Fatiguesymptomatik notwendig. Dies kann durch die Befragung des Patienten anhand einer numerischen Ratingskala (0: keine Erschöpfung; 10: stärkste vorstellbare Erschöpfung) oder durch validierte Fragebögen wie der Brief Fatigue Inventory (BFI) oder QLQ-FA12 der European Organisation for Research and Treatment of Cancer (EORTC) erfolgen. Vor Therapiebeginn mit einem PARPi ist die Durchführung einer Basisdiagnostik zur Untersuchung bereits bestehender Anämie oder Elektrolytstörungen notwendig. Organdysfunktionen, Infektionen, endokrinologische Störungen (z.B. Schilddrüsenfunktion) sowie eine Pharmakoüberdosierung, insbesondere bei morphinhaltiger Schmerzmedikation, sollten ausgeschlossen werden ( Abb. 4 ).

#### Management von Nebenwirkungen unter PARPi-Therapie

Unter PARPi ist die Fatiguebelastung besonders zu Therapiebeginn am höchsten und kann sich im Verlauf abschwächen. Bei milder bis moderater Fatigue werden nicht pharmakologische Maßnahmen empfohlen, zu denen individuell abgestimmtes körperliches Ausdauer- und Krafttraining, Verhaltenstherapie, Psychoedukation und Stressbewältigungsstrategien gehören. Die Angehörigen sollten in die Aufklärung der Patienten über dieses Symptom miteinbezogen werden. Körperliches Training ist besonders bei nicht kachektischen Patienten empfehlenswert, beispielsweise in Form von 30- bis 60-minütigen Spaziergängen 2–3-mal pro Woche oder aerobem Ausdauertraining mit 50–90% der maximalen Herzfrequenz über eine Dauer von 30–60 Minuten. Zur Verbesserung der Fatigue können Kortikosteroide kurzfristig in Erwägung gezogen werden 37 . Sollte sich dadurch keine Besserung einstellen, ist eine Therapiepause mit dem PARPi bis zum Abklingen der Symptome ratsam, gefolgt von einer Fortführung der Behandlung mit reduzierter Dosis. Besteht die Fatigue trotz adäquatem Nebenwirkungsmanagement weiterhin, ist gegebenenfalls eine Unterbrechung der PARPi-Therapie sowie eine weiterführende Abklärung indiziert ( Abb. 4 ).

### Diarrhö 3 28 39

#### Definition

**Abb. 5:**
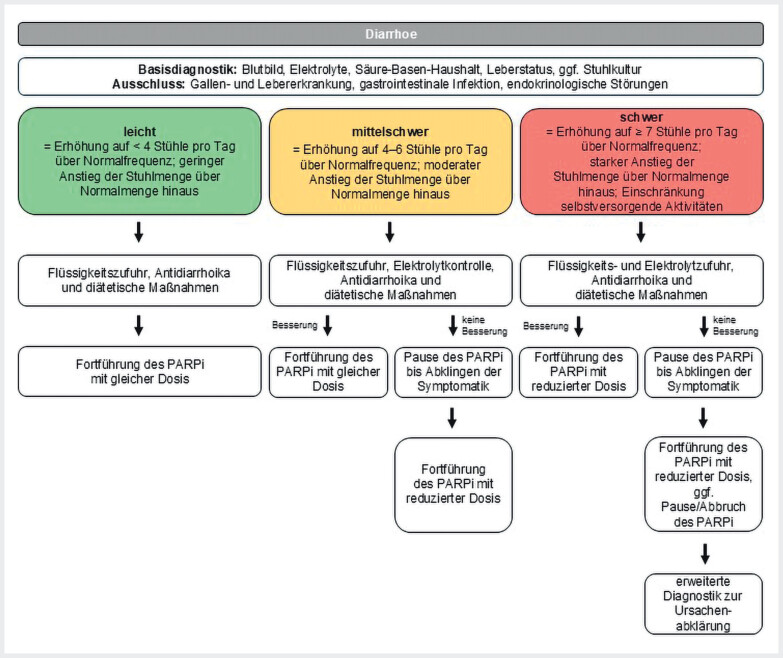
Schema zum Therapiemanagement bei Auftreten einer Diarrhö 28 30 39 . PARPi=PARP-Inhibitor

Die Diarrhö zeichnet sich durch mehr als 3 Stuhlentleerungen pro Tag sowie eine verringerte Stuhlkonsistenz aus. Die tumortherapieinduzierte Diarrhö wird in verschiedene Schweregrade unterteilt: leicht=bis zu 4 Stuhlentleerungen pro Tag, mittelschwer=4–6 Stuhlentleerungen und schwer ab 7 Stuhlentleerungen ( Abb. 5 ). Zu den Risikofaktoren für eine therapiebedingte Diarrhö zählen hohes Alter, verminderter Performance-Status, bestehende Darmdysfunktionen (z.B. chronisch entzündliche Darmerkrankungen), vorangegangene Darmoperationen mit daraus resultierender Darmdysfunktion sowie Mangelernährung.

#### Diagnostik vor Therapieeinleitung mit PARPi

Vor Beginn der Behandlung sollten der habituelle Stuhlgang einschließlich Stuhlfrequenz und -konsistenz sowie Übelkeit, Erbrechen und die Fähigkeit zur Aufnahme von Flüssigkeit und Nahrung erfasst werden. Für die Basisdiagnostik sollten Blutbild, Elektrolyte, Säure-Basen-Haushalt und Leberstatus erhoben werden. Differenzialdiagnostisch sind Gallen- und Lebererkrankungen, gastrointestinale Infektionen sowie endokrinologische Störungen abzuklären. Bei entsprechender Indikation sind laborchemische und mikrobiologische Stuhluntersuchungen durchzuführen ( Abb. 5 ). Der Nutzen einer medikamentösen Prophylaxe ist nicht nachgewiesen. Auch der Einsatz von Pro-, Prä- und Synbiotika zur Prophylaxe wird nicht empfohlen 28 .

#### Management von Nebenwirkungen unter PARPi-Therapie

Leichte Diarrhöen werden konservativ behandelt. Dies umfasst eine ausreichende orale Flüssigkeitszufuhr sowie die Gabe von Antidiarrhoika wie Loperamid. Zu Beginn wird eine Dosis von 4 mg empfohlen, gefolgt von 2 mg alle 4 Stunden oder nach jedem flüssigen Durchfall. Die Behandlung mit Loperamid sollte 12 Stunden nach dem letzten flüssigen Stuhlgang beendet werden. Zudem werden diätetische Maßnahmen empfohlen, wie der Verzicht auf laktosehaltige Produkte und hochosmolare Nahrungsergänzungsmittel. Es ist ratsam, häufiger kleinere Mahlzeiten einzunehmen. Weitere diagnostische Maßnahmen beinhalten einen Ultraschall oder CT bei zusätzlichen Schmerzen, Blutkulturen bei Fieber sowie spezielle Untersuchung auf pathogene Erreger. Bei Diarrhöen, die auf Loperamid nicht ansprechen, kann die Gabe von Octreotid (off-label) 500 µg 3-mal täglich, Tinctura opii 5–15 Tropfen 3–4-mal täglich, Budenosid 9 mg 1-mal täglich, Racecadrotril 100 mg 3-mal täglich oder oralen Aminoglykosiden in Erwägung gezogen werden 28 . Tritt durch die Maßnahmen keine Besserung ein, sollte eine Therapiepause bis Abklingen der Symptome erwogen und der PARPi mit reduzierter Dosis fortgeführt werden. Schwere Diarrhöen können eine stationäre Einweisung und intravenöse Flüssigkeits- und Elektrolytzufuhr erfordern. Besteht die Diarrhö trotz adäquatem Nebenwirkungsmanagement weiterhin, ist eine weiterführende Abklärung indiziert ( Abb. 5 ).

### Übelkeit und Erbrechen 28 40

#### Definition

Zum Symptomkomplex gehören Übelkeit, Würgereiz und Erbrechen. Bei leichter Übelkeit ist die Nahrungsaufnahme nicht beeinträchtigt. Mittelschwere Übelkeit führt zu einer eingeschränkten Nahrungsaufnahme und bei starker Übelkeit ist die Nahrungsaufnahme nahezu unmöglich. Erbrechen wird unterteilt in leicht mit 1–2 Episoden pro Tag, mittelschwer mit 3–5 Episoden und schwer mit >6 Episoden pro Tag. Die Intensität und Dauer der Symptomatik hängen von mehreren Faktoren ab, darunter die Art und Dosis der eingesetzten antineoplastischen Substanz, die Kombination mit anderen Medikamenten, eine begleitende Strahlentherapie sowie individuelle Risikofaktoren. Zu den erhöhten emetogenen Risikofaktoren zählen ein junges Alter, eine ängstliche Persönlichkeit sowie eine bekannte Vorgeschichte von Übelkeit und Erbrechen einschließlich Reisekrankheit. Grundsätzlich wird bei der oralen Tumortherapie zwischen hohem/moderatem Risiko (≥30%) und minimalem/geringem Risiko (<30%) unterschieden 41 .

#### Prophylaxe vor Therapieeinleitung mit PARPi

Bei Kombinationstherapien richtet sich die Wahl der Emesis-Prophylaxe nach der antineoplastischen Substanz mit dem höchsten emetogenen Potenzial. PARP-Inhibitoren haben ein geringes emetogenes Potenzial von unter 30%. Patienten ohne individuelle Emesis-Risikofaktoren benötigen bei allen PARPi-/ARPi-Kombinationen keine Emesis-Prophylaxe. Bei Vorliegen individueller Emesis-Risikofaktoren kann jedoch eine prophylaktische Gabe von Antiemetika erwogen werden. Mögliche Optionen sind:

- Metoclopramid oder 5-HT _3_ -Rezeptorantagonisten
- Dexamethason.

#### Management von Nebenwirkungen unter PARPi-Therapie

**Abb. 6:**
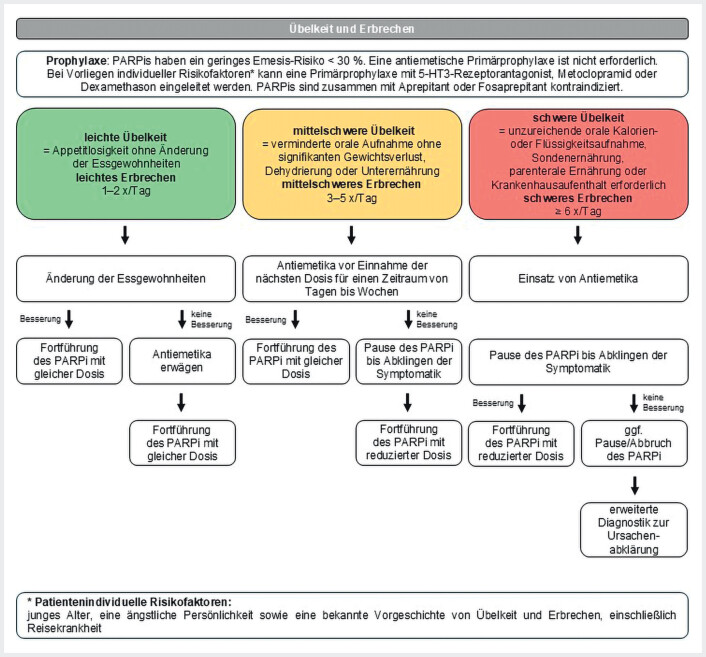
Schema zum Therapiemanagement bei Auftreten einer Übelkeit und Erbrechen 28 30 . 5-HT _3_ -Rezeptorantagonist=5-Hydroxytryptamin 3-Rezeptorantagonist, PARPi=PARP-Inhibitor

Bei leichter Übelkeit sollte zunächst eine Anpassung der Ernährungsgewohnheiten erfolgen. Falls keine Besserung eintritt, kann eine antiemetische Therapie mit Metoclopramid versucht werden. Eine Dosisreduktion der PARPi-Therapie ist in diesem Fall nicht erforderlich. Bei mäßiger bis starker Übelkeit ist eine gezielte antiemetische Behandlung indiziert. Dazu gehören Metoclopramid, 5-HT _3_ -Rezeptorantagonisten oder auch Kombinationen mit 5-HT _3_ -Rezeptorantagonisten plus Dexamethason. Die Dauer der antiemetischen Therapie variiert je nach Schweregrad der Symptome und kann wenige Tage hin bis zu mehreren Wochen erforderlich sein. Falls trotz adäquater antiemetischer Therapie keine zufriedenstellende Symptombesserung erreicht wird, sollte die PARPi-Therapie vorübergehend pausiert werden. Bei Wiederaufnahme der Behandlung kann eine Dosisreduktion erforderlich sein ( Abb. 6 ).

### Obstipation 28 42

#### Definition

**Abb. 7:**
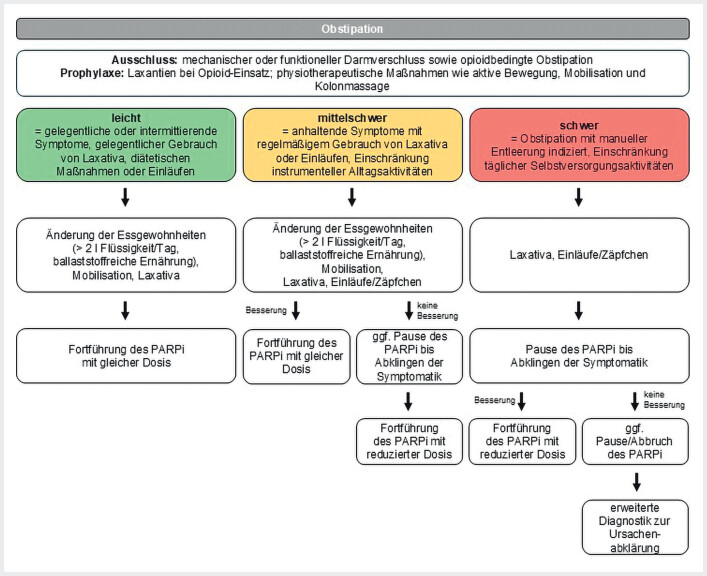
Schema zum Therapiemanagement bei Auftreten einer Obstipation 28 32 . PARPi=PARP-Inhibitor

Obstipation ist definiert als eine verringerte Stuhlfrequenz von <3-mal pro Woche mit fester Konsistenz. Die Schwere der Obstipation wird in 3 Grade unterteilt:

- Leicht: Gelegentliche oder intermittierende Symptome, die gelegentlich den Einsatz von Laxativa oder diätetischen Anpassungen erforderlich machen.
- Mittelschwer: Anhaltende Symptome, die eine regelmäßige Einnahme von Laxativa und diätetische Maßnahmen notwendig machen.
- Schwer: Obstipation, die eine manuelle Entleerung erfordert und die selbstständige Durchführung alltäglicher Aktivitäten einschränkt ( Abb. 7 ).

#### Diagnostik vor Therapieeinleitung mit PARPi und Prophylaxe

Vor Beginn der Behandlung sollte ein vorbestehender mechanischer oder funktioneller (tumorbedingter) Darmverschluss ausgeschlossen werden. Bei der Verordnung von Opioiden wird eine routinemäßige medikamentöse Prophylaxe empfohlen. Ergänzend können physiotherapeutische Maßnahmen wie aktive Bewegung, Mobilisation und Kolonmassage unterstützend eingesetzt werden ( Abb. 7 ).

#### Management von Nebenwirkungen unter PARPi-Therapie

Unabhängig vom Schweregrad der Obstipation sollte eine tägliche Flüssigkeitsaufnahme von mindestens 2 Litern angestrebt werden. Die körperliche Aktivität sollte – entsprechend den individuellen Möglichkeiten – gefördert und die Ernährung ballaststoffreich gestaltet werden. Je nach Schweregrad sind folgende Maßnahmen ratsam:

Bei leichter bis moderater Obstipation werden osmotische oder stimulierende Laxativa empfohlen, wie zum Beispiel Macrogol/Elektrolyte, Natriumpicosulfat oder Bisacodyl. Sollte die Wirkung dieser Mittel sowie von Suppositorien bei mittelschwerer Obstipation nicht ausreichen, kann eine vorübergehende Unterbrechung der PARPi-Therapie bis zur Besserung der Symptome in Erwägung gezogen werden.

Bei schwerer Obstipation sollte eine sofortige und intensivierte Behandlung mit Laxativa und Suppositorien erfolgen. Gleichzeitig wird die PARPi-Therapie pausiert, bis sich die Symptome gebessert haben. Weitere unterstützende Maßnahmen umfassen Kolonmassagen, Einläufe oder manuelle Ausräumung.

## Fazit

Die Kombination von PARPi und ARPi stellt eine vielversprechende neue Therapieoption für mCRPC-Patienten dar. Besonders Patienten mit Defekten in den HRR-Genen profitieren deutlich von der PARPi-/ARPi-Kombination. In der TALAPRO-2-Studie konnte erstmals ein signifikanter Überlebensvorteil der Kombinationstherapie sowohl für die Gesamtpopulation als auch für die HRR-positiven Patienten gezeigt werden. Der erfolgreiche Einsatz der PARPi-/ARPi-Kombination in der klinischen Praxis erfordert jedoch ein gezieltes Management von Nebenwirkungen sowie die frühzeitige Implementierung unterstützender Maßnahmen. Mit einer sorgfältigen Begleitung ist diese Therapie auch für ältere und komorbide Patienten oft gut tolerierbar und umsetzbar.

## Notes

Therapiebögen verfügbar unter
https://solution-akademie-veranstaltungen.de/systemtherapie/therapieboegen-talazoparib-enzalutamid

## Footnotes

1 Aus Gründen der besseren Lesbarkeit wird das generische Maskulinum verwendet. Entsprechende Bezeichnungen gelten im Sinne der Gleichbehandlung grundsätzlich für alle Geschlechter.

## Zusatzmaterial

Supplementary Material Zusatzmaterial

## References

[1] Chaudhuri AR Nussenzweig A The multifaceted roles of PARP1 in DNA repair and chromatin remodelling Nat Rev Mol Cell Biol 2017 18 610 621 28676700 10.1038/nrm.2017.53 PMC6591728

[2] Flippot R Patrikidou A Aldea M PARP Inhibition, a New Therapeutic Avenue in Patients with Prostate Cancer Drugs 2022 82 719 733 35511402 10.1007/s40265-022-01703-5

[3] LaRose M Manji GA Bates SE Beyond BRCA: Diagnosis and management of homologous recombination repair deficient pancreatic cancer Semin Oncol 2024 51 36 44 38171988 10.1053/j.seminoncol.2023.11.001

[4] Pezaro C PARP inhibitor combinations in prostate cancer Ther Adv Med Oncol 2020 12 1758835919897537 10.1177/1758835919897537 PMC7081465 32215055

[5] Agarwal N Zhang T Efstathiou E The biology behind combining poly [ADP ribose] polymerase and androgen receptor inhibition for metastatic castration-resistant prostate cancer Eur J Cancer 2023 192 113249 10.1016/j.ejca.2023.113249 37672815

[6] Cerrato A Morra F Celetti A Use of poly ADP-ribose polymerase (PARP) inhibitors in cancer cells bearing DDR defects: the rationale for their inclusion in the clinic J Exp Clin Cancer Res 2016 35 179 27884198 10.1186/s13046-016-0456-2 PMC5123312

[7] Javle M Curtin NJ The potential for poly (ADP-ribose) polymerase inhibitors in cancer therapy Ther Adv Med Oncol 2011 3 257 267 22084640 10.1177/1758834011417039 PMC3210467

[8] Asim M Tarish F Zecchini HI Synthetic lethality between androgen receptor signalling and the PARP pathway in prostate cancer Nat Commun 2017 8 374 28851861 10.1038/s41467-017-00393-y PMC5575038

[9] Li L Karanika S Yang G Enzalutamide-induced “BRCAness” and PARP inhibition is a synthetic lethal therapy for castration-resistant prostate cancer Sci Signal 2017 10 eaam7479 10.1126/scisignal.aam7479 PMC5855082 28536297

[10] Nizialek E Haffnwe M Bhamidipati a The effect of PARP inhibition on androgen receptor localization and activity in castration resistant prostate cancer J Clin Oncol 2022 40 e17037 e17037

[11] Schiewer MJ Goodwin JF Han S Dual roles of PARP-1 promote cancer growth and progression Cancer Discov 2012 2 1134 1149 22993403 10.1158/2159-8290.CD-12-0120 PMC3519969

[12] de Bono J Mateo J Fizazi K Olaparib for Metastatic Castration-Resistant Prostate Cancer N Engl J Med 2020 382 2091 2102 32343890 10.1056/NEJMoa1911440

[13] Agarwal N Azad AA Carles J Talazoparib plus enzalutamide in men with first-line metastatic castration-resistant prostate cancer (TALAPRO-2): a randomised, placebo-controlled, phase 3 trial Lancet 2023 402 291 303 37285865 10.1016/S0140-6736(23)01055-3

[14] Chi KM Rathkopf D Smith MR Niraparib and Abiraterone Acetate for Metastatic Castration-Resistant Prostate Cancer J Clin Oncol 2023 41 3339 3351 36952634 10.1200/JCO.22.01649 PMC10431499

[15] Clarke NW Armstrong AJ Thiery-Vuillemin A Abiraterone and Olaparib for Metastatic Castration-Resistant Prostate Cancer NEJM Evid 2022 1 EVIDoa2200043 10.1056/EVIDoa2200043 38319800

[16] Fizazi K Azad AA Matsubara N First-line talazoparib with enzalutamide in HRR-deficient metastatic castration-resistant prostate cancer: the phase 3 TALAPRO-2 trial Nat Med 2024 30 257 264 38049622 10.1038/s41591-023-02704-x PMC10803259

[17] Agarwal N Azad A Carles J Talazoparib plus enzalutamide in men with metastatic castration-resistant prostate cancer: final overall survival results from the randomised, placebo-controlled, phase 3 TALAPRO-2 trial Lancet 2025 406 447 460 40683290 10.1016/S0140-6736(25)00684-1

[18] Fizazi K Azad A Matsubara N Talazoparib plus enzalutamide in men with HRR-deficient metastatic castration-resistant prostate cancer: final overall survival results from the randomised, placebo-controlled, phase 3 TALAPRO-2 trial Lancet 2025 406 461 474 40683287 10.1016/S0140-6736(25)00683-X

[19] Chi KN Sandhu S Smith MR Niraparib plus abiraterone acetate with prednisone in patients with metastatic castration-resistant prostate cancer and homologous recombination repair gene alterations: second interim analysis of the randomized phase III MAGNITUDE trial Ann Oncol 2023 34 772 782 37399894 10.1016/j.annonc.2023.06.009 PMC10849465

[20] Saad F Clarke NW Mototsuga O Olaparib plus abiraterone versus placebo plus abiraterone in metastatic castration-resistant prostate cancer (PROpel): final prespecified overall survival results of a randomised, double-blind, phase 3 trial Lancet Oncol 2023 24 1094 1108 37714168 10.1016/S1470-2045(23)00382-0

[21] Fachinformation Akeega, Stand: Juli 2024

[22] Fachinformation Talzenna, Stand: April 2024

[23] Fachinformation Lynparza, Stand: August 2024

[24] Fachinformation Xtandi, Stand: Januar 2025

[25] Fachinformation Zytiga, Stand: Juni 2022

[26] Boelk H Yazgan SC Yekedüz E Androgen receptor pathway inhibitors and drug-drug interactions in prostate cancer ESMO Open 2024 9 103736 10.1016/j.esmoop.2024.103736 PMC11533040 39426080

[27] Gajra A Zettler M Klink AJ Acute Myeloid Leukemia/Myelodysplastic Syndrome (AML/MDS) Associated with PARP Inhibitors: A Real-World Analysis Blood 2020 136 1 19

[28] Leitlinienprogramm Onkologie (Deutsche Krebsgesellschaft, Deutsche Krebshilfe, AWMF): Konsultationsfassung S3-Leitlinie Supportive Therapie bei onkologischen PatientInnen Langversion 2.01, 2024, AWMF-Registernummer: 032–054OL https://www.leitlinienprogramm-onkologie.de/fileadmin/user_upload/LL_Supportive_Therapie_Langversion_2.01_Konsultationsfassung.pdf

[29] Onkopedia Leitlinien: Thrombozytopenie (November 2023). Zugriff am 10.04.2025 unter https://www.onkopedia.com/de/onkopedia/guidelines/thrombozytopenien/@@guideline/html/index.html

[30] Shore ND Broder MS Barata PC Expert Consensus Recommendations on the Management of Treatment-emergent Adverse Events Among Men with Prostate Cancer Taking Poly-ADP Ribose Polymerase Inhibitor + Novel Hormonal Therapy Combination Therapy Eur Urol Oncol 2025 8 94 104 38866640 10.1016/j.euo.2024.05.009

[31] Madariaga A Bowering V Ahrari S Manage wisely: poly (ADP-ribose) polymerase inhibitor (PARPi) treatment and adverse events J Gynecol Cancer 2020 30 903 915 10.1136/ijgc-2020-001288 PMC7398227 32276934

[32] Kuderer NM Dale DC Crawford J Mortality, morbidity, and cost associated with febrile neutropenia in adult cancer patients Cancer 2006 106 2258 2266 16575919 10.1002/cncr.21847

[33] Klastersky J de Naurois J Rolston K Management of febrile neutropaenia: ESMO Clinical Practice Guidelines Ann Oncol 2016 27 5 v111 v118 27664247 10.1093/annonc/mdw325

[34] Kastersky J Paesmans M Rubenstein EB The Multinational Association for Supportive Care in Cancer risk index: a multinational scoring system for identifying low-risk febrile neutropenic cancer patients J Clin Oncol 2000 18 3038 3051 10944139 10.1200/JCO.2000.18.16.3038

[35] Berger AM Mooney K Alvarez-Perez A Cancer-Related Fatigue, Version 2.2015 J Natl Compr Canc Netw 2015 13 1012 1039 26285247 10.6004/jnccn.2015.0122 PMC5499710

[36] Onkopedia Leitlinien: Fatigue (September 2018) https://www.onkopedia.com/de/ayapedia/guidelines/fatigue/@@guideline/html/index.html

[37] Fabi A Bhargava R Fatigoni S Cancer-related fatigue: ESMO Clinical Practice Guidelines for diagnosis and treatment Ann Oncol 2020 31 713 723 32173483 10.1016/j.annonc.2020.02.016

[38] Weiß J Heckl U Psychoedukation mit Krebspatienten. Hintergrund und wissenschaftliche Evidenz Der Onkologe 2021 27 54 62

[39] Bossi P Antonuzzo A Cherny NI Diarrhoea in adult cancer patients: ESMO Clinical Practice Guidelines Ann Oncol 2018 29 4 iv126 iv142 29931177 10.1093/annonc/mdy145

[40] Onkopedia Leitlinien: Antiemese bei medikamentöser Tumortherapie (Mai 2021) https://www.onkopedia.com/de/onkopedia/guidelines/antiemese-bei-medikamentoeser-tumortherapie/@@guideline/html/index.html#ID0EQOAC

[41] Hesketh P Kris MG Basch E Antiemetics: ASCO Guideline Update J Clin Oncol 2020 38 2782 2797 32658626 10.1200/JCO.20.01296

[42] Leitlinienprogramm Onkologie (Deutsche Krebsgesellschaft, Deutsche Krebshilfe, AWMF): Erweiterte S3-Leitlinie Palliativmedizin für Patienten mit einer nicht-heilbaren Krebserkrankung. Langversion 2.2, 2020, AWMF-Registernummer: 128/001OL https://www.leitlinienprogramm-onkologie.de/fileadmin/user_upload/Downloads/Leitlinien/Palliativmedizin/Version_2/LL_Palliativmedizin_Langversion_2.2.pdf

