# Supplementary material for: Therapy Management of PARP Inhibitor Combinations in mCRPC Clinical Practice
Source: Aktuelle Urol. 2025 Oct 27;56(6):552–68. [Article in German] doi: 10.1055/a-2669-8781 (PMC12646740; doi:10.1055/a-2669-8781)
Supplement: Supplementary file 1 — Supplementary Material [file 10-1055-a-2669-8781_27006244.pdf]

Thieme

Klinikum rechts der Isar   Technische Universität München

Wichtige Hinweise zur Kombinationstherapie mit Talazoparib 0.5 mg/d & Enzalutamid 160 mg/d

Aktuelle Fachinformation beachten

Indikation / Zulassung

Talazoparib wird in Kombination mit Enzalutamid zur Behandlung erwachsener Patienten mit metastasiertem kastrationsresistenten Prostatakarzinom (mCRPC) angewendet, bei denen eine Chemotherapie klinisch nicht indiziert ist.

ADT

Die Kombination mit Talazoparib & Enzalutamid wird immer unter einer Androgen-Deprivationstherapie (ADT) durchgeführt. Patienten sollten eine GnRH-Analogon- oder eine GnRH-Antagonisten Therapie erhalten. Alternativ kann eine bilaterale Orchiektomie durchgeführt werden.

Auswahl der Patienten

Für die Auswahl von mCRPC-Patienten für eine Behandlung mit Talazoparib und Enzalutamid ist kein Tumormutationstest erforderlich.

Dosierung

Die empfohlene Dosis beträgt 0,5 mg (= 2 x 0,25 mg Kps.) Talazoparib in Kombination mit 160 mg (= 4 x 40 mg Tbl. oder 2 x 80 mg Tbl.) Enzalutamid einmal täglich.

Stand der Fachinformationen: Xtandi™ Mai 2022; Talzenna® Januar 2024

©Margitta Retz, 2025. Alle Rechte vorbehalten. Dieser Therapiebogen ist urheberrechtlich geschützt. Eine Vervielfältigung, Verbreitung, Bearbeitung oder öffentliche Wiedergabe ist ohne ausdrückliche schriftliche Zustimmung des Urhebers nicht gestattet.

Talazoparib - Wichtige Informationen
**Einnahme von Talazoparib:**<br>Die empfohlene Dosis beträgt einmal täglich 0.5 mg (= 2 x 25 mg Kps.) Talazoparib als Kapsel. Die Kapsel soll unzerteilt oral eingenommen werden. Die Kapsel kann unabhängig von den Mahlzeiten eingenommen werden. Säurereduzierende Arzneimittel einschl. Protonenpumpenhemmer haben keine signifikante Auswirkung auf die Resorption von Talazoparib. Falls der Patient sich erbricht oder eine Dosis auslässt, sollte keine zusätzliche Dosis eingenommen werden. Die nächste verordnete Dosis sollte zur üblichen Zeit eingenommen werden.
**Darreichungsform:** 0,1 mg Kps. und 0,25 mg Kps.
**Terminale Halbwertszeit:** Talazoparib 90 (+/- 58) Stunden, ca. 3-6 Tage.
**Eingeschränkte Nierenfunktion:**<br>Talazoparib wird unverändert hauptsächlich über die Nieren ausgeschieden.<br>Leicht Niereninsuffizienz (CrCl 60 - 90 ml/min): Keine Dosisanpassung<br>Mittelschwere Niereninsuffizienz (CrCl 30 - 59 ml/min): Talazoparib 0.35 mg/Tag<br>Schwere Niereninsuffizienz (CrCl 15 - 29 ml/min): Talazoparib 0.25 mg/Tag<br>Es liegen keine Studiendaten für Patienten mit CrCl <15 ml/min oder bei Hämodialyse vor.
**Eingeschränkte Leberfunktion:**<br>Talazoparib wird minimal über die Leber verstoffwechselt. Bei leicher bis schwerer Leberinsuffizienz ist keine Dosisanpassung mit Talazoparib erforderlich. Die Anwendung von *Talazoparib plus Enzalutami*d wird für Patienten mit schwerer Leberinsuffizienz (Gesamtbilirubin >3 x ULN, beliebige AST) nicht empfohlen, da Pharmakokinetik und Sicherheit in dieser Gruppe nicht ermittelt wurden.
**P-Glykoprotein-Inhibitoren:**<br>Die gleichzeitige Anwendung von starken P-Glykoprotein-Inhibitoren während der Behandlung mit Talazoparib sollte vermieden werden. P-Glykoprotein-Inhibitoren führen zu einer erhöhten Talzoparib Exposition im Blut. Folgende starke P-Glykoproteine sollten vermieden werden: Amiodaron, Carvedilol, Chinidin, Ciclosporin, Clarithromycin, Cobicistat, Curcumin, Darunavir, Dronedaron, Erythromycin, Indinavir, Itraconazol, Ketoconazol, Lapatinib, Lopinavir, Propafenon, Ranolazin, Ritonavir, Saquinavir, Telaprevir, Tipranavir und Verapamil. Wenn auf die gleichzeitige Anwendung von starken P-Glykoprotein-Inhibitoren während der Behandlung mit Talazoparib nicht verzichtet werden kann, empfiehlt sich ggf. eine Dosisreduktion von Talazoparib auf 0.35 mg/Tag.
**P-Glykoprotein-Induktoren:**<br>Die gleichzeitige Anwendung von starken P-Glykoprotein-Induktoren kann die Talazoparib Exposition im Blut verringern. Rifampicin als starker P-Glykoprotein-Induktor zeigte keine Veränderungen in der Talazoparib Exposition im Blut. Allerdings wurden andere P-Glykoprotein-Induktoren nicht getestet. Folgende starke P-Glykoprotein-Induktoren sollten mit Vorsicht eingesetzt werden: Carbamazepin, Phenytoin und Johanniskraut.
**Myelodysplastisches Syndrom (MDS) /Akute myeloische Leukämie (AML):**<br>Die Gesamtinzidenz des myelodysplastischen Syndroms (MDS) bzw. der akuten myeloischen Leukämie (AML) bei Patienten mit soliden Tumoren in klinischen Studien mit Talazoparib Monotherapie betrug 0.4%. In der TALAPRO-2 Studie zeigte sich in der Kombination Talazoparib und Enzalutamid eine Inzidenz von 0.4% (2/565). Ein erhöhtes Risiko besteht bei Patienten, die zuvor eine Platin-basierte Chemotherapie und/ oder eine Strahlentherapie in der Vorgeschichte hatten. Bei Verdacht auf ein MDS oder eine AML soll eine Knochenmarksanalyse durchgeführt werden.
**Empfängnisverhütung bei Männern:**<br>Patienten müssen während und 4 Monate nach der letzten Einnahme von Talazoparib beim Geschlechtsverkehr mit Frauen im gebärfähigen Alter oder mit schwangeren Frauen ein wirksames Verhütungsmittel anwenden (auch nach Vasektomie).

©Margitta Retz, 2025. Alle Rechte vorbehalten. Dieser Therapiebogen ist urheberrechtlich geschützt. Eine Vervielfältigung, Verbreitung, Bearbeitung oder öffentliche Wiedergabe ist ohne ausdrückliche schriftliche Zustimmung des Urhebers nicht gestattet.

Klinikum rechts der Isar Technische Universität München

Enzalutamid - Wichtige Informationen
**Einnahme von Enzalutamid:** Die empfohlene Tagesgesamtdosis beträgt 160 mg (= 4 x 40 mg Tbl. oder 2 x 80 mg Tbl.) oral als tägliche Einmalgabe. Die Tabletten sollen unzerteilt als Ganzes mit Wasser oral geschluckt werden. Enzalutamid kann unabhängig von den Mahlzeiten eingenommen werden. Wenn eine Dosis ausgelassen wurde, ist diese am selben Tag so bald wie möglich nachzuholen. Wenn der Patient die Dosis über einen gesamten Tag vergessen hat, sollte die Einnahme am nächsten Tag mit der üblichen Tagesdosis fortgesetzt werden. Es dürfen keine zusätzlichen Tabletten eingenommen werden, um die versäumte Einnahme zu ersetzen.
**Die terminale Halbwertszeit** von Enzalutamid beträgt 5,8 (2,8 – 10,2) Tage.
**Eingeschränkte Nierenfunktion:** Es ist keine Dosisanpassung bei leichter bis mäßiger Nierenfunktionsstörung (Kreatinin-Clearance $\geq$ 30 ml/min) erforderlich. Es liegen keine klinischen Erfahrungen bei schwerer Nierenfunktionsstörung (Kreatinin-Clearance $\leq$ 29 ml/min) oder teminaler Niereninsuffizienz vor. Höchste Vorsicht ist geboten.
**Eingeschränkte Leberfunktion:** Es ist keine Dosisanpassung bei Leberfunktionsstörung aller Grade (Child-Pugh-Klasse A bis C) erforderlich. Bei Patienten mit schwerer Leberfunktionsstörung wurde eine verlängerte Enzalutamid Halbwertszeit beobachtet. Eine Dosisanpassung ist nicht erfoderlich.
**Sekundäre Primärtumore:** In klinischen Studien mit Enzalutamid wurden Fälle von sekundären Primärtumoren bei Patienten berichtet, die häufiger auftraten als unter Placebo. Dazu gehören Adenokarzinome des Kolons und Urothelkarzinome der Harnblase.
**Risiko von Krampfanfällen:** In klinischen Studien kam es bei 0,5% der Patienten, die mit Enzalutamid behandelt wurden, zu einem Krampfanfall. Enzalutamid und seine aktiven Metabolite binden an die GABA-aktivierten Chlorid-Kanäle und inhibieren diese Kanäle. Dadurch kann die Krampfschwelle gesenkt werden. Der Einsatz von Enzalutamid bei Patienten mit Krampfanfällen in der Vorgeschichte soll kritisch bewertet werden.
**Posteriores Reversibles Enzephalopathie-Syndrom (PRES):** Wenige Fälle mit einem PRES wurden berichtet. Aufgrund von subkortikalen Ödemen können folgende Symptome reversibel auftreten: Akute Hypertonie, epileptische Anfälle, Kopfschmerzen, Verwirrtheit, Bewusstseinsstörungen, fokale neurologische Symptome und Sehstörungen. Es wird eine MRT-Diagnostik empfohlen. Bei Nachweis eines PRES soll Enzalutamid abgesetzt werden.
**Kardiovaskuläre Risikofaktoren:** Erhöhte Vorsicht ist geboten bei Myokardinfarkt innerhalb der vergangenen 6 Monate, instabiler Angina pectoris, Herzinsuffizienz NYHA III-IV, Bradykardie und unkontrollierter Bluthochdruck.
**Überempfindlichkeitsreaktion:** Unter der Behandlung mit Enzalutamid wurden folgende Symptome beobachtet: Ödeme von Gesicht, Zunge, Lippen oder Pharynx, zusätzlich Hautausschläge und schwere kutane Nebenwirkungen.
**Fertilität und Kontrazeptiva:** Tierexperimente haben gezeigt, dass Enzalutamid das Reproduktionssystem beeinträchtigen kann. Es ist nicht bekannt, ob Enzalutamid oder seine Metabolite im Sperma vorhanden sind. Wenn ein Patient mit einer Frau im gebärfähigen Alter sexuell aktiv ist, sollte während und für 3 Monate nach Ende der Behandlung mit Enzalutamid sichere Kontrazeptiva eingesetzt werden.

©Margitta Retz, 2025. Alle Rechte vorbehalten. Dieser Therapiebogen ist urheberrechtlich geschützt. Eine Vervielfältigung, Verbreitung, Bearbeitung oder öffentliche Wiedergabe ist ohne ausdrückliche schriftliche Zustimmung des Urhebers nicht gestattet.

Enzalutamid - Wichtige Informationen
**Auswirkungen auf die Verkehrstüchtigkeit und das Bedienen von Maschinen:** Auf das potentielle Risiko eines psychischen oder neurologischen Ereignisses muss beim Führen von Fahrzeugen oder das Bedienen von Maschinen hingewiesen werden.
**Erhöhtes Monitoring bei Patienten mit Herzrhythmusstörungen:** Eine Androgendeprivationstherapie kann die QT-Zeit verlängern. Patienten mit Risikofaktoren für eine QT-Zeit-Verlängerung benötigen eine regelmäßige kardiologische Überwachung. Die gleichzeitige Gabe von Enzalutamid mit Medikamenten, die das QT-Intervall verlängern können, sollte sorgfältig überprüft werden. Zu den Medikamenten mit potentieller Wirkung auf eine QT-Intervall Verlängerung gehören: Antiarrhythmika Klasse IA (Chinidin, Disopyramid), Klasse III (Amiodaron, Sotalol, Dofetilid, Ibutilid), Methadon, Moxifloxacin, Antipsychotika wie z.B. Haloperidol.
**Starke CYP2C8-Inhibitoren erhöhen die Enzalutamid Konzentration:** CYP2C8-Enzyme spielen eine wichtige Rolle bei der Elimination von Enzalutamid. CYP2C8-Inhibitoren erhöhen den Plasmaspiegel von Enzalutamid beträchtlich und sollten daher möglichst vermieden werden. Kann auf CYP2C8-Inhibitoren nicht verzichtet werden, dann muss die Dosis von Enzalutamid auf 80 mg/Tag reduziert werden. Zu den CYP2C8-Inhibitoren gehören Clopidogrel, Cotrimoxazol, Deferasirox, Gemfibrozil, Glitazone, Leflunimid, Montelukast, Quercetin, Teriflunomid.
**Starke CYP3A4-Inhibitoren erhöhen die Enzalutamid Konzentration:** Zu den CYP3A4 Inhibitoren gehören Amiodaron, Aprepitant, Atazanavir-Ritonavir, Ciprofloxacin, Clarithromycin, Cobicistat, Crizotinib, Darunavir-Ritonavir, Diltiazem, Dronedaron, Erythromycin, Fluconazol, Fluvoxamin, Grapefruit, Idelalisib, Imatinib, Isavuconazol, Itraconazol, Ketoconazol, Lopinavir-Ritonavir, Nelfinavir, Nilotinib, Posaconazol, Ribociclib, Ritonavir, Verapamil, Voriconazol. Es ist keine Dosisanpassung erforderlich.
**CYP2C8- und CYP3A4 Induktoren erniedrigen die Enzalutamid-Konzentration:** Zu den CYP2C8- und CYP3A4 Induktoren gehören Bosentan, Carbamazepin, Johanniskraut, Mitotan, Modafinil, Nevirapin, Oxcarbazepin, Phenobarbital, Phenytoin, Primidon, Rifampicin, Rifabutin, Rifapentin. Es ist keine Dosisanpassung erforderlich.
**Enzalutamid ist ein starker Induktor von CYP3A4 & moderater Induktor von CYP2C9/CYP2C19:** Enzalutamid erniedrigt die Wirkung der folgenden Medikamente: Midazolam, S-Warfarin, Omeprazol. Die Gefahr einer Leberschädigung nach Paracetamolgabe ist bei Patienten, die gleichzeitig mit einem Enzyminduktor behandelt werden, vermutlich höher.
**Enzalutamid ist ein Inhibitor des Effluxtransporters P-Glykoprotein (P-gp):** Enzalutamid erhöht die Wirkung der folgenden Medikamente: Digoxin, Colchicin, Dabigatranetexilat. Es ist ggf. eine Dosisreduktion von insbesondere Digoxin erforderlich.
**Enzalutamid ist ein potenter Enzyminduktor und kann zu einem Verlust der Effektivität zahlreicher Arzneimittel führen:** Insbesondere sollte die gleichzeitige Behandlung mit Warfarin und Cumarin-artigen Antikoagulanzien vermieden werden. Kann auf die Antikoagulanzien nicht verzichtet werden, so müssen regelmäßige Kontrollen der INR-Werte erfolgen.

©Margitta Retz, 2025. Alle Rechte vorbehalten. Dieser Therapiebogen ist urheberrechtlich geschützt. Eine Vervielfältigung, Verbreitung, Bearbeitung oder öffentliche Wiedergabe ist ohne ausdrückliche schriftliche Zustimmung des Urhebers nicht gestattet.

Enzalutamid - Wichtige Informationen	
**Enzalutamid kann die Wirkung der folgenden Arzneimittel vermindern:**	
Analgetika	Fentanyl, Tramadol
Antibiotika	Clarithromycin, Doxycyclin
Zytostatika	Cabazitaxel
Antiepileptika	Carbamazepin, Clonazepam, Phenytoin, Valproinsäure
Antipsychotika	Haloperidol
Antithrombotika	Acenocumarol, Warfarin, Clopidogrel
Betablocker	Bisoprolol, Propranolol
Ca-Antagonisten	Diltiazem, Felodipin, Nicardipin, Nifedipin, Verapamil
Herzglykoside	Digoxin
Kortikosteroide	Dexamethason, Prednisolon
Antivirale HIV-Arzneimittel	Indinavir, Ritonavir
Hypnotika	Diazepam, Midazolam, Zolpidem
Immunsuppressiva	Tacrolimus
Protonenpumpenhemmer	Omeprazol
Statine	Atorvastatin, Simvastatin
Schilddrüsenhormone	Levothyroxin

Nebenwirkungen – Kombination Talazoparib, Enzalutamid und ADT laut Fachinformation

Häufigste Nebenwirkungen >10% aller Toxizitätsgrade laut Talazoparib Fachinformation:

- Anämie, Neutropenie, Leukopenie und Thrombopenie
- Verminderter Appetit, Übelkeit, Erbrechen, Dysgeusie, Stomatitis
- Diarrhoe, Obstipation
- Fatigue, Schwindel, Kopfschmerzen
- Alopezie
- Venöse Thromboembolie

Häufigste hämatotoxische Nebenwirkungen ≥Grad 3 in der TALAPRO-2 Studie:

- Anämie 46%
- Neutropenie 18%
- Thrombozytopenie 7%

Ereignisse infolge von Nebenwirkungen mit der Kombination Talazoparib, Enzalutamid & ADT:

- Therapiepausierung 62% (Häufigste NW Anämie)
- Dosisreduktion 53% (Häufigste NW Anämie)
- Therapieabbruch 19% (Häufigste NW Anämie)

Insgesamt waren bei 42,5% der Patienten Bluttransfusionen erforderlich.

Hämatologische Toxizitäten:

Vor Behandlungsbeginn sollten die Parameter des Blutbildes ≤ CTCAE Grad 1 sein:

- Hämoglobin (≥ 10.0 g/dL)
- Neutrophile (≥ 1500 /ul)
- Thrombozyten (≥ 75000 /ul)

Eine Untersuchung des Differentialblutbildes vor Therapiebeginn und nachfolgende monatliche Kontrollen werden für die ersten 12 Behandlungsmonate sowie nach medizinischer Indikation empfohlen. Sollte ein Patient eine schwerwiegende hämatologische Toxizität entwickeln und Bluttransfusionen benötigen, sollte die Behandlung mit Talazoparib unterbrochen werden. Wenn die Blutparameter auch nach einer vierwöchigen Unterbrechung der Behandlung mit Talazoparib klinisch abnormal bleiben, werden eine Analyse des Knochenmarks und/ oder eine zytogenetische Blutanalyse empfohlen.

©Margitta Retz, 2025. Alle Rechte vorbehalten. Dieser Therapiebogen ist urheberrechtlich geschützt. Eine Vervielfältigung, Verbreitung, Bearbeitung oder öffentliche Wiedergabe ist ohne ausdrückliche schriftliche Zustimmung des Urhebers nicht gestattet.

Nebenwirkungen mit Talazoparib<br>Basierend auf einem gepoolten Datensatz aus 8 Studien (n=1088)			
	**Grad 1 - 4 (%)**	**Grad 3 (%)**	**Grad 4 (%)**
**Bösartige Neubildungen**			
MDS/ Akute myeloische Leukämie	**0.2**	**<0,1**	**<0,1**
**Erkrankungen des Blutes und Lymphsystems**			
Anämie	**55,6**	**37,8**	**1,5**
Neutropenie	**30,3**	**15,0**	**1,6**
Thrombozytopenie	**25,2**	**8,1**	**3,0**
Leukopenie	**17,9**	**4,8**	**0,2**
Lymphopenie	**8,1**	**3,4**	**0,4**
**Ernährungsstörungen**			
Verminderter Appetit	**21,1**	**1,0**	**0**
**Erkrankungen des Nervensystems**			
Kopfschmerz	**19,0**	**0,7**	**-**
Schwindel	**14,4**	**0,4**	**< 0,1**
Dysgeusie	**6,3**	**0**	**0**
**Gefäßerkrankungen**			
Venöse Thromboembolie	**3,3**	**2,1**	**0,2**
**Erkrankungen des Gastrointestinaltraktes**			
Übelkeit	**35,8**	**0,9**	**-**
Diarrhoe	**18,8**	**0,4**	**0**
Erbrechen	**15,3**	**0,8**	**0**
Abdominalschmerz	**14,9**	**1,1**	**-**
Dyspepsie	**6,3**	**0**	**-**
Stomatitis	**5,0**	**0**	**0**
**Erkrankungen der Haut**			
Alopezie	**17,4**	**-**	**-**
**Allgemeine Beschwerden**			
Fatigue	**52,5**	**5,3**	**-**

Management von Nebenwirkungen mit der Kombination Talazoparib		
**Nebenwirkungen**	Pausierung Talazoparib bis Erreichen folgender Werte	Wiederaufnahme von Talazoparib
Hämoglobin < 8 g/dl	≥ 9 g/dl	Wiederaufnahme der Behandlung mit Talazoparib mit der nächstniedrigen Dosierung.
Thrombozyten < 50.000/ul	Thrombozyten ≥ 75.000/ul	
Neutrophile < 1000/ul	Neutrophile ≥ 1500/ul	
Nicht-hämatologische NW Grad 3 oder 4	≤ Grad 1	Wiederaufnahme der Behandlung mit Talazoparib mit Dosisreduktion oder endgültig absetzen.
**Empfohlene Anfangsdosis: Talazoparib 0.5 mg/ Tag**<br>1. Dosisreduktion = **0.35 mg/Tag**; 2. Dosisreduktion = **0.25 mg/Tag**; 3. Dosisreduktion = **0.1 mg/Tag**		
**Management von Nebenwirkungen mit der Kombination Enzalutamid**		
Nicht-hämatologische NW Grad 3 oder 4	≤ Grad 2	Wiederaufnahme der Behandlung mit Enzalutamid und eventuell Dosisreduktion auf 120 mg/Tag.

©Margitta Retz, 2025. Alle Rechte vorbehalten. Dieser Therapiebogen ist urheberrechtlich geschützt. Eine Vervielfältigung, Verbreitung, Bearbeitung oder öffentliche Wiedergabe ist ohne ausdrückliche schriftliche Zustimmung des Urhebers nicht gestattet.

Klinikum rechts der Isar Technische Universität München

Arzt - Dokumentation
Talazoparib & Enzalutamid

Patientenaufkleber

Indikation / Zulassung

Talazoparib wird in Kombination mit Enzalutamid zur Behandlung erwachsener Patienten mit metastasiertem kastrationsresistenten Prostatakarzinom (mCRPC) angewendet, bei denen eine Chemotherapie klinisch nicht indiziert ist.

Empfohlene Tagesdosis nach Fachinformation			
	morgens	mittags	abends
**Talazoparib 0,25 mg Kps.**<br>Unabhängig von den Mahlzeiten	**2**	0	0
**Enzalutamid 40 mg Tbl.**<br>**oder**<br>**Enzalutamid 80 mg Tbl.**<br>Unabhängig von den Mahlzeiten	**4**<br><br>**2**	0<br><br>0	0<br><br>0

©Margitta Retz, 2025. Alle Rechte vorbehalten. Dieser Therapiebogen ist urheberrechtlich geschützt. Eine Vervielfältigung, Verbreitung, Bearbeitung oder öffentliche Wiedergabe ist ohne ausdrückliche schriftliche Zustimmung des Urhebers nicht gestattet.

Talazoparib & Enzalutamid

Klinikum rechts der Isar   Technische Universität München

**Arztdokumentation<br>Vor Einleitung der Kombination Talazoparib und Enzalutamid**		
**Patientenname**	**Datum:**	
**Anamnese**	ja	nein
Patientenaufklärung vor Therapiebeginn		
Hämoglobin $\geq$ 10 g/dl		
Neutrophile $\geq$ 1,5 x 10^9 /l		
Thrombozyten $\geq$ 75 x 10^9 /l		
Elektrolyte im Normbereich		
Arterielle Hypertonie		
Kardiovaskuläre Erkrankungen		
Störungen der Knochenmarksfunktion		
Störungen des Gastrointestinaltraktes		
Störungen der Leberfunktion		
Störungen der Nierenfunktion		
Störungen der Atemwege		
Störungen des Nervensystems		
Hauterkrankungen		
Sonstiges:		
Allergien:		
Hormonablation erfolgt (GnRH-Analoga/-Antagonisten oder Orchiektomie)		

**Untersuchungen**			Check ✔
Blutdruck	Puls	ECOG	
EKG-Befund			
Ggf. Herzecho-Befund			
Sonografie Nieren		Restharn	
Wichtige klinische Untersuchungsbefunde:			

**Labor**	
Differential-Blutbild	
Natrium, Kalium, Kalzium, Magnesium, Kreatinin, Phosphat, Harnstoff, LDH, GOT, GPT, Bilirubin, Alkalische Phosphatase	
Urin-Stix, ggf. Mikrobiologie	

**Medikamentenliste**	

©Margitta Retz, 2025. Alle Rechte vorbehalten. Dieser Therapiebogen ist urheberrechtlich geschützt. Eine Vervielfältigung, Verbreitung, Bearbeitung oder öffentliche Wiedergabe ist ohne ausdrückliche schriftliche Zustimmung des Urhebers nicht gestattet.

Talazoparib & Enzalutamid

Klinikum rechts der Isar   Technische Universität München

Medikamenten-Anamnese: Vor Einleitung der Talazoparib-Therapie			
**Folgende Medikamente können die Wirkung von Talazoparib signifikant erhöhen. Eine Dosisreduktion von Talazoparib ist erforderlich. Die reduzierte Talazoparib Tagesdosis beträgt 0.35 mg Kps.**			
	✔		✔
Amiodaron		Ketoconazol	
Carvedilol		Lapatinib	
Chinidin		Lopinavir	
Ciclosporin		Propafenon	
Clarithromycin		Ranolazin	
Cobicistat		Ritonavir	
Darunavir		Saquinavir	
Dronedaron		Telaprevir	
Erythromycin		Telithromycin	
Indinavir		Tipranavir	
Itraconazol		Verapamil	
Keine Konsumierung von Grapefruitsaft und Curcumin (Gelbwurz/Kurkumawurzel)			
**Folgende Medikamente können die Wirkung von Talazoparib erniedrigen. Die gleichzeitige Gabe der aufgelisteten Medikamente mit Talazoparib sollte vermieden werden.**			
	✔		✔
Carbamazepin		Phenytoin	
Keine Konsumierung von Johanniskraut			

Medikamenten-Anamnese: Vor Einleitung der Enzalutamid-Therapie			
**Folgende Medikamente können die Wirkung von Enzalutamid erhöhen. Die gleichzeitige Gabe der aufgelisteten Medikamente mit Enzalutamid sollte vermieden werden. Die reduzierte Enzalutamid Tagesdosis beträgt 80 mg/Tag.**			
	✔		✔
Cotrimoxazol		Leflunimid	
Clopidogrel		Montelukast	
Gemfibrozil		Quercetin	
Glitazone		Teriflunomid	
**Folgende Medikamente können die Wirkung von Enzalutamid erniedrigen. Die gleichzeitige Gabe der aufgelisteten Medikamente mit Enzalutamid sollte vermieden werden.**			
	✔		✔
Bosentan		Phenobarbital	
Carbamazepin		Phenytoin	
Mitotan		Primidon	
Modafinil		Rifampicin	
Nevirapin		Rifabutin	
Oxcarbazepin		Rifapentin	
Keine Konsumierung von Johanniskraut			

©Margitta Retz, 2025. Alle Rechte vorbehalten. Dieser Therapiebogen ist urheberrechtlich geschützt. Eine Vervielfältigung, Verbreitung, Bearbeitung oder öffentliche Wiedergabe ist ohne ausdrückliche schriftliche Zustimmung des Urhebers nicht gestattet.

Medikamenten-Anamnese: Vor Einleitung der Enzalutamid-Therapie			
**Enzalutamid kann die Wirkung der folgenden Medikamente erhöhen. Die gleichzeitige Gabe der aufgelisteten Medikamente mit Enzalutamid sollte vermieden werden.**			
	✔		✔
Digoxin		Dabigatranetexilat	
Colchicin			
**Enzalutamid kann die Wirkung der folgenden Medikamente erniedrigen. Die gleichzeitige Gabe der aufgelisteten Medikamente mit Enzalutamid sollte vermieden werden.**			
	✔		✔
Midazolam		Omeprazol	
S-Warfarin			

Enzalutamid ist ein potenter Enzyminduktor und kann zu einem Verlust der Effektivität der folgenden Medikamente führen.	
Analgetika	Fentanyl, Tramadol
Antibiotika	Clarithromycin, Doxycyclin
Zytostatika	Cabazitaxel
Antiepileptika	Carbamazepin, Clonazepam, Phenytoin, Valproinsäure
Antipsychotika	Haloperidol
Antithrombotika	Acenocumarol, Warfarin, Clopidogrel
Betablocker	Bisoprolol, Propranolol
Ca-Antagonisten	Diltiazem, Felodipin, Nicardipin, Nifedipin, Verapamil
Herzglykoside	Digoxin
Kortikosteroide	Dexamethason, Prednisolon
Antivirale HIV-Arzneimittel	Indinavir, Ritonavir
Hypnotika	Diazepam, Midazolam, Zolpidem
Immunsuppressiva	Tacrolimus
Protonenpumpenhemmer	Omeprazol
Statine	Atorvastatin, Simvastatin
Schilddrüsenhormone	Levothyroxin

Erhöhtes Monitoring bei Patienten mit Herzrhythmusstörungen
Eine Androgendeprivationstherapie kann die QT-Zeit verlängern. Patienten mit Risikofaktoren für eine QT-Zeit-Verlängerung benötigen regelmäßige kardiologische Kontrollen. Die gleichzeitige Gabe von Enzalutamid mit Medikamenten, die das QT-Intervall verlängern können, sollte sorgfältig überprüft werden. Folgende Medikamente können potentiell das QT-Intervall verlängern:
Antiarrhythmika Klasse IA: Chinidin, Disopyramid
Antiarrhythmika Klasse III: Amiodaron, Sotalol, Dofetilid, Ibutilid
Opioid: Methadon
Antibiotika: z.B. Moxifloxacin
Antipsychotika: z.B. Haloperidol

©Margitta Retz, 2025. Alle Rechte vorbehalten. Dieser Therapiebogen ist urheberrechtlich geschützt. Eine Vervielfältigung, Verbreitung, Bearbeitung oder öffentliche Wiedergabe ist ohne ausdrückliche schriftliche Zustimmung des Urhebers nicht gestattet.

Talazoparib & Enzalutamid

Klinikum rechts der Isar   Technische Universität München

Management von Nebenwirkungen mit der Kombination Talazoparib		
**Nebenwirkungen**	Pausierung von Talazoparib bis zum Erreichen folgender Werte	Wiederaufnahme von Talazoparib
Hämoglobin < 8 g/dl	≥ 9 g/dl	Wiederaufnahme der Behandlung mit Talazoparib mit der nächstniedrigen Dosierung.
Thrombozyten < 50.000/ul	Thrombozyten ≥ 75.000/ul	
Neutrophile < 1000/ul	Neutrophile ≥ 1500/ul	
Nicht-hämatologische NW Grad 3 oder 4	≤ Grad 1	Wiederaufnahme der Behandlung mit Talazoparib mit der nächstniedrigen Dosierung in Betracht ziehen oder Talazoparib endgültig absetzen.

Empfohlene Anfangsdosis: Talazoparib 0.5 mg/ Tag

Dosisreduktionen von Talazoparib: 1. Dosisreduktion = 0.35 mg/Tag
2. Dosisreduktion = 0.25 mg/Tag
3. Dosisreduktion = 0.1 mg/Tag

Management von Nebenwirkungen mit der Kombination Enzalutamid:		
Nicht-hämatologische NW Grad 3 oder 4	≤ Grad 2	Wiederaufnahme der Behandlung mit Enzalutamid und ggf. Dosisreduktion auf 120 mg/Tag.

Zusätzliche Notizen:

©Margitta Retz, 2025. Alle Rechte vorbehalten. Dieser Therapiebogen ist urheberrechtlich geschützt. Eine Vervielfältigung, Verbreitung, Bearbeitung oder öffentliche Wiedergabe ist ohne ausdrückliche schriftliche Zustimmung des Urhebers nicht gestattet.

Talazoparib & Enzalutamid

Klinikum rechts der Isar
Technische Universität München

**Arztdokumentation<br>Kombination Talazoparib & Enzalutamid – Kontrolluntersuchung Nr. 1**		
**Wichtig: Kontrolle des Differentialblutbildes mindestens alle 4 Wochen!**		
**Patientenname**	**Datum:**	
**Anamnese**	ja	nein
Hämoglobin ≥ 9 g/dl		
Neutrophile > 1,5 x 10^9 /l		
Thrombozyten > 75 x 10^9 /l		
Elektrolyte im Normbereich		
Arterielle Hypertonie		
Kardiovaskuläre Erkrankungen		
Stomatitis, Geschmacksstörungen, verminderter Appetit, Übelkeit oder Erbrechen		
Diarrhoe oder Obstipation		
Störungen der Leberfunktion		
Störungen der Nierenfunktion		
Störungen der Atemwege		
Störungen des Nervensystems		
Schwindel, Kopfschmerzen		
Hauterkrankungen		
Fatigue		
Sonstiges:		
Hormonablation fortgeführt (GnRH-Analoga/-Antagonisten oder Orchiektomie)		

**Untersuchungen**			Check ✔
Blutdruck	Puls	ECOG	
Ggf. EKG-Befund			
Ggf. Herzecho-Befund			
Ggf. Sonografie Nieren		Restharn	
Besonderheiten:			

**Labor**	
Differential-Blutbild	
Natrium, Kalium, Kalzium, Magnesium, Kreatinin, Phosphat, Harnstoff, LDH, GOT, GPT, Bilirubin, Alkalische Phosphatase	
Urin-Stix, ggf. Mikrobiologie	

**Medikamentenliste**	**morgens**	**mittags**	**abends**
**Talazoparib ………… mg Kps.**			
**Enzalutamid ………….mg Tbl.**			

©Margitta Retz, 2025. Alle Rechte vorbehalten. Dieser Therapiebogen ist urheberrechtlich geschützt. Eine Vervielfältigung, Verbreitung, Bearbeitung oder öffentliche Wiedergabe ist ohne ausdrückliche schriftliche Zustimmung des Urhebers nicht gestattet.

Klinikum rechts der Isar

Technische Universität München

Arztdokumentation
Kombination Talazoparib & Enzalutamid – Kontrolluntersuchung Nr. 2

Wichtig: Kontrolle des Differentialblutbildes mindestens alle 4 Wochen!

**Patientenname**	**Datum:**

**Anamnese**	ja	nein
Hämoglobin $\geq$ 9 g/dl		
Neutrophile > 1,5 x 10^9 /l		
Thrombozyten > 75 x 10^9 /l		
Elektrolyte im Normbereich		
Arterielle Hypertonie		
Kardiovaskuläre Erkrankungen		
Stomatitis, Geschmacksstörungen, verminderter Appetit, Übelkeit oder Erbrechen		
Diarrhoe oder Obstipation		
Störungen der Leberfunktion		
Störungen der Nierenfunktion		
Störungen der Atemwege		
Störungen des Nervensystems		
Schwindel, Kopfschmerzen		
Hauterkrankungen		
Fatigue		
Sonstiges:		
Hormonablation fortgeführt (GnRH-Analoga/-Antagonisten oder Orchiektomie)		

**Untersuchungen**			Check ✔
Blutdruck	Puls	ECOG	
Ggf. EKG-Befund			
Ggf. Herzecho-Befund			
Ggf. Sonografie Nieren		Restharn	
Besonderheiten:			

**Labor**	
Differential-Blutbild	
Natrium, Kalium, Kalzium, Magnesium, Kreatinin, Phosphat, Harnstoff, LDH, GOT, GPT, Bilirubin, Alkalische Phosphatase	
Urin-Stix, ggf. Mikrobiologie	

**Medikamentenliste**	**morgens**	**mittags**	**abends**
**Talazoparib ………… mg Kps.**			
**Enzalutamid ………….mg Tbl.**			

©Margitta Retz, 2025. Alle Rechte vorbehalten. Dieser Therapiebogen ist urheberrechtlich geschützt. Eine Vervielfältigung, Verbreitung, Bearbeitung oder öffentliche Wiedergabe ist ohne ausdrückliche schriftliche Zustimmung des Urhebers nicht gestattet.

Thieme

Klinikum rechts der Isar

Technische Universität München

Arztdokumentation<br>Kombination Talazoparib & Enzalutamid – Kontrolluntersuchung Nr. 3		
**Wichtig: Kontrolle des Differentialblutbildes mindestens alle 4 Wochen!**		
**Patientenname**	**Datum:**	
**Anamnese**	ja	nein
Hämoglobin ≥ 9 g/dl		
Neutrophile > 1,5 x 10^9 /l		
Thrombozyten > 75 x 10^9 /l		
Elektrolyte im Normbereich		
Arterielle Hypertonie		
Kardiovaskuläre Erkrankungen		
Stomatitis, Geschmacksstörungen, verminderter Appetit, Übelkeit oder Erbrechen		
Diarrhoe oder Obstipation		
Störungen der Leberfunktion		
Störungen der Nierenfunktion		
Störungen der Atemwege		
Störungen des Nervensystems		
Schwindel, Kopfschmerzen		
Hauterkrankungen		
Fatigue		
Sonstiges:		
Hormonablation fortgeführt (GnRH-Analoga/-Antagonisten oder Orchiektomie)		

**Untersuchungen**			Check ✔
Blutdruck	Puls	ECOG	
Ggf. EKG-Befund			
Ggf. Herzecho-Befund			
Ggf. Sonografie Nieren		Restharn	
Besonderheiten:			

**Labor**	
Differential-Blutbild	
Natrium, Kalium, Kalzium, Magnesium, Kreatinin, Phosphat, Harnstoff, LDH, GOT, GPT, Bilirubin, Alkalische Phosphatase	
Urin-Stix, ggf. Mikrobiologie	

**Medikamentenliste**	**morgens**	**mittags**	**abends**
**Talazoparib ………… mg Kps.**			
**Enzalutamid ………….mg Tbl.**			

©Margitta Retz, 2025. Alle Rechte vorbehalten. Dieser Therapiebogen ist urheberrechtlich geschützt. Eine Vervielfältigung, Verbreitung, Bearbeitung oder öffentliche Wiedergabe ist ohne ausdrückliche schriftliche Zustimmung des Urhebers nicht gestattet.

Talazoparib & Enzalutamid

Klinikum rechts der Isar Technische Universität München

Arztdokumentation<br>Kombination Talazoparib & Enzalutamid – Kontrolluntersuchung Nr. 4		
**Wichtig: Kontrolle des Differentialblutbildes mindestens alle 4 Wochen!**		
**Patientenname**	**Datum:**	
**Anamnese**	ja	nein
Hämoglobin $\geq$ 9 g/dl		
Neutrophile > 1,5 x 10^9 /l		
Thrombozyten > 75 x 10^9 /l		
Elektrolyte im Normbereich		
Arterielle Hypertonie		
Kardiovaskuläre Erkrankungen		
Stomatitis, Geschmacksstörungen, verminderter Appetit, Übelkeit oder Erbrechen		
Diarrhoe oder Obstipation		
Störungen der Leberfunktion		
Störungen der Nierenfunktion		
Störungen der Atemwege		
Störungen des Nervensystems		
Schwindel, Kopfschmerzen		
Hauterkrankungen		
Fatigue		
Sonstiges:		
Hormonablation fortgeführt (GnRH-Analoga/-Antagonisten oder Orchiektomie)		

**Untersuchungen**			Check ✔
Blutdruck	Puls	ECOG	
Ggf. EKG-Befund			
Ggf. Herzecho-Befund			
Ggf. Sonografie Nieren		Restharn	
Besonderheiten:			

**Labor**	
Differential-Blutbild	
Natrium, Kalium, Kalzium, Magnesium, Kreatinin, Phosphat, Harnstoff, LDH, GOT, GPT, Bilirubin, Alkalische Phosphatase	
Urin-Stix, ggf. Mikrobiologie	

**Medikamentenliste**	**morgens**	**mittags**	**abends**
**Talazoparib ………… mg Kps.**			
**Enzalutamid ………….mg Tbl.**			

©Margitta Retz, 2025. Alle Rechte vorbehalten. Dieser Therapiebogen ist urheberrechtlich geschützt. Eine Vervielfältigung, Verbreitung, Bearbeitung oder öffentliche Wiedergabe ist ohne ausdrückliche schriftliche Zustimmung des Urhebers nicht gestattet.

Technische Universität München

Patienten-Tagebuch

Talazoparib & Enzalutamid

Patientenaufkleber

Indikation / Zulassung

Talazoparib wird in Kombination mit Enzalutamid zur Behandlung erwachsener Patienten mit metastasiertem kastrationsresistenten Prostatakarzinom (mCRPC) angewendet, bei denen eine Chemotherapie klinisch nicht indiziert ist.

Tägliche Dokumentation der
Tabletteneinnahmen, Blutdruck, Temperatur und Nebenwirkungen
Frühzeitige Benachrichtigung des Arztes bei Nebenwirkungen!

Empfohlene Tagesdosis nach Fachinformation			
	morgens	mittags	abends
**Talazoparib 0,25 mg Kps.**<br>Unabhängig von den Mahlzeiten	**2**	0	0
**Enzalutamid 40 mg Tbl.**<br>**oder**	**4**	0	0
**Enzalutamid 80 mg Tbl.**<br>Unabhängig von den Mahlzeiten	**2**	0	0

©Margitta Retz, 2025. Alle Rechte vorbehalten. Dieser Therapiebogen ist urheberrechtlich geschützt. Eine Vervielfältigung, Verbreitung, Bearbeitung oder öffentliche Wiedergabe ist ohne ausdrückliche schriftliche Zustimmung des Urhebers nicht gestattet.

Patienten-Tagebuch

Talazoparib & Enzalutamid

Technische Universität München

Wichtige Informationen für den Patienten
**Wie nehme ich Talazoparib ein?**<br>Die empfohlene Tagesgesamtdosis beträgt 0.5 mg (2 x 0,25 mg Kps.) als tägliche Einmalgabe. Die Kapseln sollen unzerteilt oral eingenommen werden. Talazoparib kann unabhängig von den Mahlzeiten eingenommen werden.
**Wie nehme ich Enzalutamid ein?**<br>Die empfohlene Tagesgesamtdosis beträgt 160 mg (4 x 40 mg Tbl. *oder* 2 x 80 mg Tbl.) als tägliche Einmalgabe. Die Tabletten sollen unzerteilt oral eingenommen werden. Enzalutamid kann unabhängig von den Mahlzeiten eingenommen werden.
**Wie verhalte ich mich, wenn ich eine Einnahme vergessen habe?**<br>Wenn eine Dosis ausgelassen wurde, ist diese am selben Tag so bald wie möglich nachzuholen. Wenn der Patient die Dosis über einen gesamten Tag vergessen hat, sollte die Einnahme am nächsten Tag mit der üblichen Tagesdosis fortgesetzt werden. Es dürfen keine zusätzlichen Tabletten eingenommen werden, um die versäumte Einnahme zu ersetzen.
**Was ist vor Einnahme von Talazoparib & Enzalutamid zu beachten?**<br>Talazoparib und Enzalutamid haben zahlreiche Wechselwirkungen mit anderen Medikamenten. Es ist wichtig, dass der behandelnde Arzt Ihre vollständige Medikamentenliste überprüft.
**Was sind die häufigsten Nebenwirkungen von Talazoparib und Enzalutamid?**<br>• Blutarmut, verminderte Zahl an weißen Blutkörperchen und Blutplättchen<br>• Verminderter Appetit, Übelkeit, Erbrechen<br>• Geschmacksstörungen, Mundschleimhautentzündung<br>• Durchfall, Verstopfung<br>• Müdigkeit, Schwindel, Kopfschmerzen<br>• Haarverlust<br>• Venöse Thromboembolie
**Wichtiger Hinweis:**<br>Bei Nebenwirkungen oder bei veränderten Laborwerten kann eine Dosisreduktion oder eine Pausierung von Talazoparib und/ oder Enzalutamid erforderlich sein. Der behandelnde Arzt wird mit Ihnen die notwendigen Maßnahmen besprechen.

Ich wurde ausführlich über die Verabreichung bzw. Einnahme von Talazoparib und Enzalutamid aufgeklärt. Ich habe den Medikamentenplan verstanden und alle Fragen wurden ausreichend beantwortet. Bei Nebenwirkungen werde ich mich frühzeitig bei meinem zuständigen Fachpersonal melden.

Patient (Datum, Unterschrift) **Arzt** (Datum, Unterschrift)

©Margitta Retz, 2025. Alle Rechte vorbehalten. Dieser Therapiebogen ist urheberrechtlich geschützt. Eine Vervielfältigung, Verbreitung, Bearbeitung oder öffentliche Wiedergabe ist ohne ausdrückliche schriftliche Zustimmung des Urhebers nicht gestattet.

Patienten-Tagebuch

Talazoparib & Enzalutamid

Klinikum rechts der Isar

Technische Universität München

**Patientendokumentation**	
**Patientenname:**	
**Woche:**	**Datum:**

**Medikamentenliste**	**morgens**	**mittags**	**abends**
Talazoparib ________ mg Kps.			
Enzalutamid ________mg Tbl.			

**Datum**	**Einnahme von Medikamenten**		**Wie fühlen Sie sich heute?**			**Nebenwirkungen oder Besonderheiten**	**Blutdruck**	**Temp.**
	morgens ✔	abends ✔	☺	😐	☹			

Bemerkungen 1:	
Bemerkungen 2:	
Gewicht nach Ende der Woche:	

©Margitta Retz, 2025. Alle Rechte vorbehalten. Dieser Therapiebogen ist urheberrechtlich geschützt. Eine Vervielfältigung, Verbreitung, Bearbeitung oder öffentliche Wiedergabe ist ohne ausdrückliche schriftliche Zustimmung des Urhebers nicht gestattet.

Akt Urol | DOI 10.1055/a-2669-8781 | © The Author(s).

Patienten-Tagebuch

Talazoparib & Enzalutamid

Klinikum rechts der Isar

Technische Universität München

Patientendokumentation	
**Patientenname:**	
**Woche:**	**Datum:**

Medikamentenliste	morgens	mittags	abends
Talazoparib ________ mg Kps.			
Enzalutamid ________mg Tbl.			

Datum	Einnahme von Medikamenten		Wie fühlen Sie sich heute?			Nebenwirkungen oder Besonderheiten	Blutdruck	Temp.
	morgens ✔	abends ✔	☺	😐	☹			

Bemerkungen 1:
Bemerkungen 2:
Gewicht nach Ende der Woche:

©Margitta Retz, 2025. Alle Rechte vorbehalten. Dieser Therapiebogen ist urheberrechtlich geschützt. Eine Vervielfältigung, Verbreitung, Bearbeitung oder öffentliche Wiedergabe ist ohne ausdrückliche schriftliche Zustimmung des Urhebers nicht gestattet.

Akt Urol | DOI 10.1055/a-2669-8781 | © The Author(s).

Patienten-Tagebuch

Talazoparib & Enzalutamid

Klinikum rechts der Isar

Technische Universität München

**Patientendokumentation**	
**Patientenname:**	
**Woche:**	**Datum:**

**Medikamentenliste**	**morgens**	**mittags**	**abends**
Talazoparib ________ mg Kps.			
Enzalutamid ________mg Tbl.			

**Datum**	**Einnahme von Medikamenten**		**Wie fühlen Sie sich heute?**			**Nebenwirkungen oder Besonderheiten**	**Blutdruck**	**Temp.**
	morgens ✔	abends ✔	☺	😐	☹			

Bemerkungen 1:
Bemerkungen 2:
Gewicht nach Ende der Woche:

©Margitta Retz, 2025. Alle Rechte vorbehalten. Dieser Therapiebogen ist urheberrechtlich geschützt. Eine Vervielfältigung, Verbreitung, Bearbeitung oder öffentliche Wiedergabe ist ohne ausdrückliche schriftliche Zustimmung des Urhebers nicht gestattet.

Akt Urol | DOI 10.1055/a-2669-8781 | © The Author(s).

Thieme

Patienten-Tagebuch

Talazoparib & Enzalutamid

Klinikum rechts der Isar

Technische Universität München

**Patientendokumentation**	
**Patientenname:**	
**Woche:**	**Datum:**

**Medikamentenliste**	**morgens**	**mittags**	**abends**
Talazoparib ________ mg Kps.			
Enzalutamid ________mg Tbl.			

**Datum**	**Einnahme von Medikamenten**		**Wie fühlen Sie sich heute?**			**Nebenwirkungen oder Besonderheiten**	**Blutdruck**	**Temp.**
	morgens ✔	abends ✔	☺	😐	☹			

Bemerkungen 1:

Bemerkungen 2:

Gewicht nach Ende der Woche:

©Margitta Retz, 2025. Alle Rechte vorbehalten. Dieser Therapiebogen ist urheberrechtlich geschützt. Eine Vervielfältigung, Verbreitung, Bearbeitung oder öffentliche Wiedergabe ist ohne ausdrückliche schriftliche Zustimmung des Urhebers nicht gestattet.

Akt Urol | DOI 10.1055/a-2669-8781 | © The Author(s).

Patienten-Tagebuch

Talazoparib & Enzalutamid

Klinikum rechts der Isar

Technische Universität München

**Patientendokumentation**	
**Patientenname:**	
**Woche:**	**Datum:**

**Medikamentenliste**	**morgens**	**mittags**	**abends**
Talazoparib ________ mg Kps.			
Enzalutamid ________mg Tbl.			

**Datum**	**Einnahme von Medikamenten**		**Wie fühlen Sie sich heute?**			**Nebenwirkungen oder Besonderheiten**	**Blutdruck**	**Temp.**
	morgens ✔	abends ✔	☺	😐	☹			

Bemerkungen 1:
Bemerkungen 2:
Gewicht nach Ende der Woche:

©Margitta Retz, 2025. Alle Rechte vorbehalten. Dieser Therapiebogen ist urheberrechtlich geschützt. Eine Vervielfältigung, Verbreitung, Bearbeitung oder öffentliche Wiedergabe ist ohne ausdrückliche schriftliche Zustimmung des Urhebers nicht gestattet.

Akt Urol | DOI 10.1055/a-2669-8781 | © The Author(s).

Thieme

Patienten-Tagebuch

Talazoparib & Enzalutamid

Klinikum rechts der Isar

Technische Universität München

**Patientendokumentation**	
**Patientenname:**	
**Woche:**	**Datum:**

**Medikamentenliste**	**morgens**	**mittags**	**abends**
Talazoparib ________ mg Kps.			
Enzalutamid ________mg Tbl.			

**Datum**	**Einnahme von Medikamenten**		**Wie fühlen Sie sich heute?**			**Nebenwirkungen oder Besonderheiten**	**Blutdruck**	**Temp.**
	morgens ✔	abends ✔	☺	😐	☹			

Bemerkungen 1:

Bemerkungen 2:

Gewicht nach Ende der Woche:

©Margitta Retz, 2025. Alle Rechte vorbehalten. Dieser Therapiebogen ist urheberrechtlich geschützt. Eine Vervielfältigung, Verbreitung, Bearbeitung oder öffentliche Wiedergabe ist ohne ausdrückliche schriftliche Zustimmung des Urhebers nicht gestattet.

Akt Urol | DOI 10.1055/a-2669-8781 | © The Author(s).

Patienten-Tagebuch

Talazoparib & Enzalutamid

Klinikum rechts der Isar   Technische Universität München

**Patientendokumentation**	
**Patientenname:**	
**Woche:**	**Datum:**

**Medikamentenliste**	**morgens**	**mittags**	**abends**
Talazoparib ________ mg Kps.			
Enzalutamid ________mg Tbl.			

**Datum**	**Einnahme von Medikamenten**		**Wie fühlen Sie sich heute?**			**Nebenwirkungen oder Besonderheiten**	**Blutdruck**	**Temp.**
	morgens ✔	abends ✔	☺	😐	☹			

Bemerkungen 1:	
Bemerkungen 2:	
Gewicht nach Ende der Woche:	

©Margitta Retz, 2025. Alle Rechte vorbehalten. Dieser Therapiebogen ist urheberrechtlich geschützt. Eine Vervielfältigung, Verbreitung, Bearbeitung oder öffentliche Wiedergabe ist ohne ausdrückliche schriftliche Zustimmung des Urhebers nicht gestattet.

Akt Urol | DOI 10.1055/a-2669-8781 | © The Author(s).

Thieme

Patienten-Tagebuch

Talazoparib & Enzalutamid

Klinikum rechts der Isar

Technische Universität München

**Patientendokumentation**	
**Patientenname:**	
**Woche:**	**Datum:**

**Medikamentenliste**	**morgens**	**mittags**	**abends**
Talazoparib ________ mg Kps.			
Enzalutamid ________mg Tbl.			

**Datum**	**Einnahme von Medikamenten**		**Wie fühlen Sie sich heute?**			**Nebenwirkungen oder Besonderheiten**	**Blutdruck**	**Temp.**
	morgens ✔	abends ✔	☺	😐	☹			
Bemerkungen 1:								
Bemerkungen 2:								
Gewicht nach Ende der Woche:								

©Margitta Retz, 2025. Alle Rechte vorbehalten. Dieser Therapiebogen ist urheberrechtlich geschützt. Eine Vervielfältigung, Verbreitung, Bearbeitung oder öffentliche Wiedergabe ist ohne ausdrückliche schriftliche Zustimmung des Urhebers nicht gestattet.

Akt Urol | DOI 10.1055/a-2669-8781 | © The Author(s).
